# Optimisation of electrospinning parameters to successfully obtain high ratios of medium chain length polyhydroxyalkanoate in electrospun fibres with drug loading for wound healing applications

**DOI:** 10.1007/s10856-026-07030-5

**Published:** 2026-03-26

**Authors:** Robyn A. Macartney, Annabelle T. R. Fricker, Gusti U. N. Tajalla, Andrew M. Smith, Shosei Kishida, Stefano Fedele, Ipsita Roy, Jonathan C. Knowles

**Affiliations:** 1 Division of Biomaterials and Tissue Engineering, University College London (UCL) Eastman Dental Institute, London, United Kingdom; 2 School of Chemical, Materials & Biological Engineering, Faculty of Engineering, University of Sheffield, Sheffield, United Kingdom; 3 Materials and Metallurgical Engineering Department, Institut Teknologi Kalimantan, Balikpapan, Indonesia; 4 Department of Microbial Diseases, UCL Eastman Dental Institute, Royal Free Campus, University College London, London, United Kingdom; 5 Department of Biochemistry and Genetics, Kagoshima University Graduate School of Medical and Dental Sciences, Kagoshima, Japan; 6 UCL Eastman Dental Institute, University College London, London, United Kingdom; 7 NIHR UCLH Biomedical Research Centre, London, UK; 8 Insigneo Institute, University of Sheffield, Sheffield, United Kingdom

## Abstract

Chronic wounds, burns and ulceration of dermal and mucosal tissues are extremely common and can arise for a wide variety of reasons causing extreme pain and reducing patient quality of life. Current treatment regimens involve the use of topical corticosteroids for prolonged treatment periods. Due to issues surrounding the use of topical ointments there is inadequate drug contact with the wound site and non-specific tissue interaction, potentially leading to significant development of fungal infections as a side effect to corticosteroid treatment. Medium chain length (MCL) and short chain length (SCL) polyhydroxyalkanoates (PHAs) may be applicable to optimise material properties for wound dressing applications. Initial work focussed on defining the optimal electrospinning parameters for suitably elastic fibres whilst subsequent work focussed on achieving an optimised dosing of clobetasol propionate (CP) and fluconazole (FLU) for incorporation with the electrospun fibres without detrimentally compromising the properties of the scaffolds for wound healing applications. Physical and mechanical analysis showed that the 80:20 blend of MCL:SCL polymer at an electrospinning solution concentration of 10% (w/v) gave defect-free fibres with the best elastic properties for wound dressing applications. CP and FLU incorporation into the electrospun fibres did not cause any significant decrease in oral mucosal cell viability. Following in vitro wound healing study promising formulations containing 2% and 10% CP and FLU, respectively, were identified.

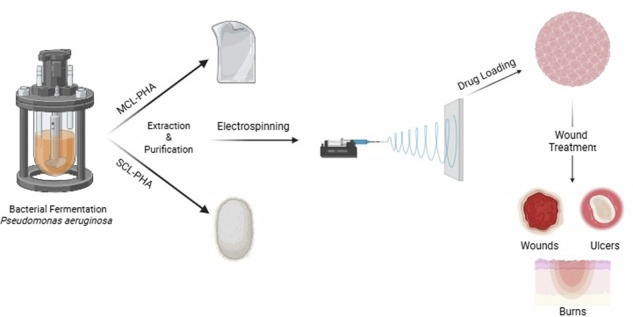

## Introduction

Healing of chronic wounds, burns and ulcers poses significant strain on healthcare systems globally. These ailments massively impact the quality of life of patients for extended periods of time, with consequences including physical pain, infection, tissue death, reduced function, and emotional and financial impacts. Common treatment regimens involve regular cleaning and covering using dressings and bandages for protection. Some wounds such as persistent oral inflammatory sores with excessive inflammation and chronic ulcers benefit from treatment with corticosteroid creams, the active pharmaceuticals include clobetasol propionate, betamethasone dipropionate, and clobetasone butyrate [1]. Topical ointment and mouthwash formulations used for these oral applications have several shortcomings, the residence time at the site of application is typically very short, they can be time consuming and messy to apply and treatment regimens can be complicated especially in cases with multiple different prescriptions. All of these concerns with topical treatments are heightened when used in the oral cavity due to salivary washout and unintended ingestion of the ointments. Additionally, secondary complications often become apparent after long-term treatments with corticosteroids, these include skin atrophy, and increased risk of infections due to topical steroids impairing local immune responses. One commonly observed secondary complication of treatment with corticosteroids is the development of oral candida a fungal infection. Therefore, corticosteroid treatment is often coupled with treatment using antifungal and antibacterials to decrease infection risk. Commonly prescribed pharmaceuticals for this purpose are nystatin, fluconazole, terbinafine and clotrimazole [2]. Within this research we propose to investigate the optimal dosing of intraoral patches containing CP or FLU for application in patients suffering these afflictions. This will provide localised and sustained release of corticosteroid (CP) at the site of injury to minimise interactions with non-target tissues and prevent problems with salivary washout. The hydrophobic nature of CP lends itself to electrospinning with hydrophobic polymers, such as the PHAs described here due to compatibility of the solvent systems. The FLU patches are intended to deal with any secondary fungal infection as a result of CP treatment. Whilst FLU is characterised as a hydrophilic drug it’s interaction with hydrophobic carriers to improve the drugs delivery, stability and efficacy in vivo has been documented. In the case of nanofibres this drug can be encapsulated within the fibres to provoke a controlled, site-specific drug release. In the past decade many research groups have shown interest in the use of electrospun materials [3–5] for use in wound care and oral transmucosal drug delivery applications. The nanofibrous, non-woven meshes created via electrospinning have many properties which make them ideal for this application. Tailorable fibre diameters allow the materials to mimic the physical structure of the natural ECM and promote the creation of high surface area to volume ratios, which promote tissue haemostasis [6, 7]. These highly porous structures are ideal for gas permeation and prevent wound dehydration, whilst the interconnectivity of the pores prevents microbial infiltration and cell ingrowth [8]. Finally, the properties of these materials can be tailored to match the mechanical strength and elasticity of natural soft tissues [9, 10]. Typically, these fibres are polymer based and depending on the specific application may be synthetic, natural or blends of both. Synthetic polymers are easily tailored to provide many different functional properties, they are strong, cheap, reliable and can be easily processed using a wide range of different methods. Unfortunately, they also have some limitations, such as the release of toxic degradation products, stimulation of local and systemic immune responses and changes in mechanical properties during early degradation. Conversely, natural polymers tend to present native biomolecular cues which can enhance biocompatibility of the materials, and some natural polymers have even been reported to have inherent antimicrobial properties, e.g. chitosan. However, natural polymers have problems with batch-to-batch variability, difficulty in harvesting and purification and risk denaturation during these processes.

Polyhydroxyalkanoates (PHAs) are a sustainable class of biocompatible, biodegradable polymers which can be produced during bacterial fermentation. These polymers are produced as an energy reserve when bacteria are grown under stressful, nutrient limiting conditions and can be extracted and purified for use in a range of fields including medical materials, pharmaceuticals, cosmetics and tissue engineering [11]. There are two main classes of PHAs, short chain length (SCL) and medium chain length (MCL), with classification depending on the number of carbons present in the monomer unit. The physical and mechanical properties of PHAs can be tailored to fit many different applications by altering the production conditions to obtain different molecular weights and number of carbon units in the monomer structure. Many different species are reportedly capable of producing PHAs including: *Ralstonia* [12], *Staphylococcus* [13], *Pseudomonas* [14, 15], and *Methylobacterium* [16, 17]. The most reported feedstock for PHA production is glucose, however as advancements are made in this polymer production technique many more options for feedstocks have become available. These include organic acids [18, 19], alcohols [20] and lipids [21–23]. The emergence of these alternative carbon sources to act as feedstock has brought the potential to use waste products of industrial processes, such as crude oil and food waste [21, 24]. Evidence shows that this is a scalable process and following optimisations documented in the literature many methodologies now give high yields of purified PHAs. This improves the financial viability of scale-up processes for this sustainable polymer source. Additionally, the emergence of the use of waste products of other industrial processes as feedstocks for the fermentations further enhances the potential for scalability of this production method [25–27].

PHAs have been electrospun in the past by various groups, showing potential for many medical applications such as cartilage tissue engineering [28], neural applications [29, 30], cardiovascular and vascular care [31]. Volova et al. investigated the effect of several parameters on the properties of electrospun SCL-PHAs. Polymer concentrations of 2–8% (w/v) in chloroform could be electrospun to obtain smooth, defect-free fibres. However, these fibres consisting solely of P(3HB) presented stiff mechanical properties with Young’s modulus of 356.23 MPa and maximum elongation at break of only 13.3% [32]. These mechanical properties are not suitable for applications such as wound dressings or soft tissue engineering where a degree of elasticity is required to ensure that the materials can withstand anatomical stresses [32]. Similar results were obtained by a different group, who also provided evidence that the mechanical properties can be tailored by changing the molar percentage of monomers present in the PHAs. The addition of different molar ratios of P(4HB) and P(3HV) to the polymer composition allowed some improvements in the mechanical properties for soft tissue applications. The best formulation for this application appeared to be the electrospun P(3HB-35.6%-co-4HB-3.4%3HV) giving a much lower Young’s modulus of 73 MPa [33]. Whilst this is significantly reduced compared to the pure P(3HB) it is still very high compared to the Young’s modulus of 1 MPa which is reported for native soft tissues [34]. MCL-PHAs are known to have much more elastic properties than SCL-PHAs. Hence, the electrospinning of MCL-PHAs has been investigated. It is found that MCL-PHAs are not electrospinnable unless blended with SCL-PHAs, ratios of at least 65% SCL-PHA are reported as being necessary for the production of discrete, homogenous fibres [35]. Using this blend ratio Young’s modulus of 34.36 MPa could be obtained [35]. Similar ratios have been used elsewhere to produce morphologically acceptable electrospun fibres [28–30]. Here, we aim to investigate the potential to electrospin SCL:MCL blends with a higher amount of MCL-PHA to provide a wider range of mechanical properties, enabling these materials to be used for a wider range of applications in wound care, soft tissue engineering and drug delivery. The industrialisation of the electrospinning processes has become much more realistic in recent years following the development of multi-needle injection systems, needleless electrospinning and roll-to-roll collectors. Initial breakthroughs in electrospinning manufacturing were in fact hindered by the lack of scalability, but these new techniques in the field now show great promise in textiles, pharmaceutical and filtration industries [36, 37].

Drug delivery is a rapidly expanding field of study and the development of new mechanisms of drug delivery provides the opportunity for sustained and targeted treatments. This brings with it a range of advantages such as less frequent administration, stable drug absorption, better patient compliance and fewer side effects [38]. Electrospinning, among other techniques such as 3D printing, microfluidics, nanoimprinting and layer-by-layer synthesis, have become popular in the manufacture of materials for drug delivery. In recent years, the functionalisation of electrospun fibres to enhance wound healing and tissue repair has been extensively explored. The inclusion of growth factors [39], antimicrobials [38], essential oils [40], and other therapeutic agents [41] in electrospun fibres has helped to enhance the wound healing potential of polymer-based dressings. In this study, we aim to optimise the SCL:MCL-PHA ratios to obtain the best mechanical properties possible for soft tissue repair and wound care applications without compromising the fibre integrity, homogeneity and morphology. Additionally, we investigate the ideal dosing of a commonly prescribed corticosteroid (clobetasol propionate (CP)) and anti-fungal (fluconazole (FLU)) within the optimised electrospun PHA fibres for future application to the treatment of oral mucosal ulcers. An intraoral patch delivery system would offer localised, sustained drug delivery with fewer side effects and improved patient compliance.

## Materials and methods

### Materials

CP powder, ≥98%, was purchased from Cambridge Bioscience (Cambridge, England, UK). FLU powder, 98%, phosphate-buffered saline tablets (PBS), chloroform, ≥99.5%, dimethylformamide, ≥99.5%, methanol (MeOH), trifluoracetic acid (TFA) and acetonitrile (ACN) were procured from Fisher Scientific (Loughborough, England, UK). Defined keratinocyte SFM, TrypLE express, MTT reagent, and Penicillin-Streptomycin, 10,000 U.mL^−1^ were obtained from Thermo Fisher Scientific (Life Technologies) (Massachusetts, US).

### Production of PHAs

#### MCL-PHA production

A Gram-negative bacterium (commercially sensitive) was streaked on isolation agar from a glycerol stock and incubated at 30 °C overnight. A 50 mL inoculum medium was prepared in a 250 mL conical flask, autoclaved, and supplemented with MgSO₄·7H₂O, a trace element solution, and a propriety substrate mixture (150 µL total). A single colony was inoculated into the medium and incubated at 30 °C with shaking at 200 rpm for 24 h. For scale-up, 400 mL of the same medium was prepared in 2 L flasks and inoculated with the overnight culture, followed by incubation under the same conditions. A sterilized 15-L bioreactor was then prepared for a 23-h fed-batch fermentation. After the fermentation, cells were harvested by centrifugation at 9000 rpm for 20 min. The supernatant was discarded, and the cell pellets were transferred to trays and stored at −80 °C for at least 1 h before freeze-drying. Freeze-drying was continued until complete moisture removal. We understand that the commercial sensitivity of gram-negative strains used in this production method cause limitations to the reproducibility of our study. Nonetheless, we have described the cultivation parameters and experimental protocols in full to facilitate replication with related PHA-producing Gram-negative species available commercially.

#### SCL-PHA production

SCL-PHA was produced using a Gram-negative bacterium, which is commercially sensitive information. However, the protocol used was very similar to that used by Basnett et al. 2021. A seed culture was prepared by inoculating a single colony into sterile nutrient broth and incubating for 16 h at 30 °C and 200 rpm. Ten percent (v/v) of this inoculum was transferred into 10 L of media containing glucose, (NH₄)₂SO₄, yeast extract, and trace elements solutions in a 15-L fermenter, operated at 30 °C, 200 rpm stirring, and 1 vvm aeration. After 48 h of cultivation, the culture broth was harvested, centrifuged at 5000 rpm for 30 min at 4 °C, and the resulting cell pellets were collected, transferred to a beaker, and stored at −20 °C for further processing.

#### Extraction and purification

Purification was performed by initially washing freeze-dried samples with methanol at room temperature overnight, a step repeated several times to ensure complete removal of residual fatty acids. The polymer was subsequently extracted with chloroform at a 1:10 (w/v) ratio and filtered through cellulose thimbles repeatedly until the supernatant was colourless. The extract was then concentrated using a rotary evaporator and precipitated with cold methanol. The resulting purified PHA was collected and dried in a desiccator.

#### Polymer characterisation

The detailed properties of the MCL-PHA produced in this study is commercially sensitive. However, they are comparable to previously reported results by S.M.D. Syed Mohamed et al., who used Pseudomonas mendocina CH50 [42]. The MCL-PHA primarily consisted of 3-hydroxydecanoate (3HD, 71.5 ± 2.4 mol%) and 3-hydroxyoctanoate (3HO, 24.4 ± 3.6 mol%), with minor fractions of 3-hydroxyhexanoate (3HHx), 3-hydroxy-dodecanoate (3HDD), and 3-hydroxy-tetradecanoate (3HTD). The polymer exhibited a weight-average molecular mass (Mw) of 461 kDa, a number-average molecular mass (Mn) of 223 kDa, and a polydispersity index (PDI) of 2.1. Meanwhile, the properties of SCL-PHA have been previously reported by Basnett et al., showing the production of 100% P(3HB) with an Mw of 458 kDa, Mn of 110 kDa, and a PDI of 4.1 [43].

### Optimisation of electrospinning parameters

MCL:SCL PHA electrospinning solutions were prepared in CHCl_3_/DMF (90/10 vol%), due to the good solubility of both MCL and SCL PHAs in CHCl_3,_ with the DMF added to slow the solvent evaporation at the needle tip and prevent clogging of the needle. The SCL-PHA was dissolved at 40 °C for 3 h under constant stirring, the solution allowed to cool and then the MCL-PHA added. The solutions were prepared at total polymer concentrations of 5 and 10% (w/v) with weight compositional ratios between the MCL:SCL of 90:10, 80:20 and 75:25. These solutions were then stirred continuously overnight on a magnetic stirring plate at room temperature.

The electrospinning set-up consisted of a high-voltage power supply (max output of 30 kV), a syringe pump and grounded, flat collector plate, arranged in a horizontal configuration with a 15 cm distance between the needle and collector. A static, flat collector is used in this study as random fibre alignment is acceptable for the application of this research. It is well documented that the use of aligned fibres yields anisotropic mechanical properties with the ductility, strength and modulus being highly dependent on the direction of loading [44–46]. Given that the materials produced here are to be used directly by untrained end users as wound dressings it is more appropriate to have the isotropic properties provided by randomly aligned fibres, so the mechanical success of the material is not dependent on the direction of application by the user. The polymer solution was transferred to a 3 mL syringe and placed in the syringe pump; the positive voltage supply was connected to the needle tip (21 G, blunt tip). All electrospinning was conducted under ambient conditions, at a temperature of 21–24 °C and recorded relative humidities of 25–35%.

Throughout these optimisation experiments 3 independent electrospinning batches were produced for each of the investigated parameters and screened using light microscopy and SEM (representative images shown in Supplementary 1, 2).

### Production of drug-loaded fibres

For the investigation of drug loading using CP and FLU the optimised electrospinning parameters were used employing a 10% (w/v) polymer concentration of 80:20 MCL:SCL, applied voltage of 20 kV and flow rate of 1 mL.hr^−1^. Drugs were suspended in 1 mL of CHCl_3_ and stirred before combining with the polymer solution, this was then stirred until homogenously distributed, concentrations reported are in relation to the weight of polymer in the solution and from here on are named as follows in Table 1. The concentrations of drug loading were decided based on the recommended therapeutic doses of currently prescribed topical treatments. CP Is most routinely prescribed in an ointment at 0.05% in addition to consultation with doses used in similar drug delivery systems in the literature [47]. The FLU dosing was decided based on the same rationale [48–50].

**Table 1:** Summarising the sample names of the control and drug-loaded samples

Drug incorporation	Sample name	Concentration of drug (w/w) to polymer in solution
None	ES_CON	N/A
Clobetasol propionate	ES_1CP	1%
Clobetasol propionate	ES_2CP	2%
Clobetasol propionate	ES_4CP	4%
Fluconazole	ES_5FLU	5%
Fluconazole	ES_10FLU	10%

### Characterisation of electrospun fibres

#### Scanning electron microscopy

All images were collected using the Sigma 300 VP system (Zeiss, Germany), in secondary electron mode using an accelerating voltage of 5 kV, at a working distance of 8 mm. Using Image-Pro v11, physical characteristics of the materials such as fibre diameter were quantified. Prior to SEM imaging, samples were deposited with a thin layer Au-Pd (~13 nm) using an Emitech K500X sputtering system (Quorum Technologies, UK) at 25 mA for 90 s.

#### FTIR

FTIR spectra for native and drug-loaded electrospun samples were obtained using the PerkinElmer Spectrum One spectrometer (PerkinElmer Ltd., Beaconsfield, UK) equipped with an attenuated total reflectance (ATR) (Golden Gate ATR, Specac Ltd., Orpington, UK) with diamond window. Samples were analysed in the 4000–600 cm^−1^ wavenumber region, at a resolution of 2 cm^−1^. The data presented in spectra was normalised by maximum point in the Spectrum software v 5.0.1 (PerkinElmer Ltd., Beaconsfield, UK).

#### Surface wettability

Water contact angle was measured to analyse surface wettability of the control and drug-loaded electrospun scaffolds. Contact angle was measured using the sessile drop method using an automated contact angle goniometer (CAM200, KSV Instruments). A 5 µL droplet of deionised water was formed and randomly chosen spots on the samples using a motor-driven syringe and the values reported is an average of 5 measurements made on different samples.

#### Dynamic mechanical analysis

Dynamic mechanical analysis was performed using the TA Instruments, DMA 850 (Delaware, US), the mechanical analysis was conducted under 3 different conditions; 1) dry conditions at room temperature, 2) dry conditions at 37 °C and 3) submerged conditions (PBS) at 37 °C. These different testing conditions were used to ensure appropriate mechanical properties during manufacture and storage, application and residence time as the patch will be stored under dry, ambient conditions, whilst application is in a moist environment at body temperature of 37 °C. Specimens were cut to dimensions of 50 × 5 × 0.1 mm (lxwxh) and stored in a desiccator prior to testing.

#### Differential scanning calorimetry

The DSC thermograms (equilibrated with an indium standard; each sample weighing 5–10 mg) were obtained during heat-cool-heat cycles using the DSC 25 system by TA Instruments (Delaware, US). The first heat cycle was obtained from 24 °C to 200 °C at a rate of 10 °C.min^−1^, the cooling cycle from 200 °C to −50 °C at a rate of 5 °C.min^−1^, the second heating cycle was obtained from −50 °C to 200 °C at a rate of 10 °C.min^−1^, all under nitrogen purge (50 ml.min^−1^).

#### Drug release studies

Drug release studies were performed in PBS dissolution medium under sink conditions at 37.0 ± 0.5 °C in a shaking incubator set to 100 rpm. Maximum solubility of CP and FLU under these conditions was determined experimentally, details are included in Supplementary 11 and 12, respectively. Maximum solubility of CP and FLU was determined as 4.498 µg.mL^−1^ and 1.287 mg.mL^−1^. For drug release studies of CP scaffolds a mass of close to 20 mg (range from 15 to 24 mg) and for FLU scaffolds a mass of close to 50 mg (range 41–57 mg) of all scaffold formulations were weighed in triplicate and immersed into vials containing 10 mL of medium and 1 mL samples were withdrawn at specific time points (0.5, 1, 2, 3, 4, 5, 6, 8, 12, and 24 h), followed by the replenishment of the withdrawn medium volume with fresh release media. For drug release samples All sample groups were analysed in triplicate. Both drugs are quantified in each withdrawn volume using the following HPLC method.

HPLC analysis was conducted using an Agilent Technologies 1100 Infinity compact LC Series (Agilent Technologies, Cheshire, UK), with a Zorbax LC column (5 μm particle size, C18, 170 Å, 250 × 4.6 mm). The separation of analytes occurred at 24 °C, utilising mobile phases composed of 0.1% TFA in water and 0.1% TFA in ACN. Conditions were as follows; 35% of ACN in water for 5 min at a flow rate of 0.6 mL.min^−1^, the flow rate was then increased to 1 ml.min^−1^ between 5 and 6 min, finally the mobile phase composition was ramped to 100% ACN (+0.1% TFA) between 5 and 15 min at 1 mL.min^−1^. The conditions were then held as such until 20 min. UV signal was measured using an Agilent G1315A DAD detector at 250 nm. For each concentration, single injections of 20 μL were analysed [51]. Calibration curves showed good linearity with R^2^ of 0.997 and 0.999 and gradients of the calibration curves were 60.23 and 1325.7 for CP and FLU, respectively. The linear ranges of calibration curves were suitable for the analyses conducted here. CP linear range was found between 0 and 5 µg.mL^−1^ and FLU linear range was found between 0 and 1000 µg.mL^−1^.

#### Loading efficiency study

To determine the maximum drug loading of all scaffold formulations, ~10 mg of each formulation was weighed and placed inside SpectraPor® regenerated cellulose dialysis tubing from Repligen (MWCO 14 kDa), which is compatible with halogenated hydrocarbons such as chloroform, with 1 mL chloroform and the sample was fully dissolved. The samples were then dialysed against chloroform overnight at room temperature. The resulting release media were then dried and resuspended in PBS for HPLC analysis. The method used is appropriate for analysis of the loading efficiency as there is no trace of the polymer left in the analysed solution, the polymer is contained within the dialysis tubing as MW was confirmed as 461 kDa and 458 kDa for the MCL and SCL polymers, respectively. The molecular weight cut off of the dialysis tubing is 14 kDa allowing the CP and FLU of molecular weights 466.97 Da and 306.27 Da, respectively, to dialyse appropriately. The analysed solution is from the external solution to the tubing, which contains only the CP and FLU as solutes.

The HPLC method previously described in Section 3.5.5 was used to quantify the concentration of CP or FLU in each respective sample group. The experiment was run in triplicate and values reported are calculated as indicated in Eq. (1).

1 $${\boldsymbol{Loading}}\,{\boldsymbol{Efficiency}}\left( \% \right)=\frac{{\boldsymbol{Calculated}}\,{\boldsymbol{Mass}}\,{\boldsymbol{of}}\,{\boldsymbol{API}}\,{\boldsymbol{Present}}\,({\boldsymbol{mg}})}{{\boldsymbol{Theoretical}}\,{\boldsymbol{Mass}}\,{\boldsymbol{of}}\,{\boldsymbol{API}}\,{\boldsymbol{Added}}\,({\boldsymbol{mg}})}\times {\bf{100}}$$

Equation (1) Calculation performed to obtain the loading efficiency for each formulation.

### Biological analysis

#### General cell culture

Mouth ordinary epithelial cells (MOE-1a) [52] were gifted by the lab of Professor Shosei Kishida and Andrew M. Smith and maintained under normal cell culture conditions (5% CO_2_, 37 °C) in defined keratinocyte serum-free media (KSFM) supplemented with the KSFM growth supplement and 1% penicillin-streptomycin. Cells were maintained at 70–80% confluence, using 1X TrypLE express for passaging procedures. These cells were used due to their similarity to the native human oral epithelial cells, expressing key epithelial markers, having intact barrier properties and are a good model for healthy oral epithelium for in vitro evaluation.

#### Cell viability

Cell viability was analysed using 3-(4,5-dimethylthiazol-2-yl)-2,5- diphenyl tetrazolium bromide (MTT). For this study, MOE-1a cells were seeded at a density of 5 × 10^4^ cells.mL^−1^, in 96 well plates and incubated at 37 °C, 5% CO_2_ and allowed to attach overnight for 12 h. Samples were washed in 1X PBS and fresh media introduced, alongside the electrospun samples (control, CP loaded, and FLU loaded) plates were then incubated for 1, 3 and 7 days with media aspirated and replaced at day 3. Positive control samples (cells cultured in complete growth media with no electrospun patch or drug treatment), control electrospun samples with no drug loading, drug controls (media with free drug dissolved) and negative controls, lysed with triton x-100 were included at each time point. The concentration for the drug control groups of CP and FLU was chosen as they are reflective of the therapeutic dosage of these drugs when prescribed in their topical form. CP control was set at 10 µg.mL^−1^ corresponding to therapeutic topical ointments, which are prescribed at a concentration of 0.01–0.05% and FLU control set at 5 mg.mL^−1^ corresponding to the concentration of 0.5% found in topical FLU-containing ointments. For the viability study, the indirect transwell setup was chosen to ensure mechanistic clarity, this setup separates the material from the cells but allows soluble factors to diffuse. Therefore, the cytotoxicity caused by released drug or leachables from the scaffold are analysed rather than the direct effect of physical contact with the cells. MTT solution (5 mg.mL^−1^ in PBS, filter sterilised) was added to a final concentration of 0.5 mg.mL^−1^ and incubated at 37 °C until the formazan crystals could be observed visually. Culture media was removed, 100 μL of solubilisation buffer added (20% w/v SDS prepared in a 1:1 ratio of DMF: deionised water, pH adjusted to 4.7 using acetic acid). Samples were briefly agitated in the dark until complete solubilisation of the formazan crystals. Following solubilisation, absorbance was read at 572 nm using a microplate reader (Tecan, Switzerland).

#### Wound healing assay

The wound healing assay was carried out using the 4-well silicone culture inserts available from Ibidi (catalogue number – 80469). Inserts were placed in 12-well plates and cells seeded at 2.5 × 10^5^ cells.mL^−1^ with 100 µL per well. Plates were incubated overnight until confluent before removal of the silicone inserts. Samples were washed gently in PBS and complete growth media was added to the wells, with patches in experimental groups placed on top of the media giving an indirect contact assay, to provide the following groups:

- Positive Control, complete growth media.
- Drug Control, CP or FLU-containing media.
- Control Scaffold, complete growth media and native ES patch.
- Loaded Scaffolds, complete growth media and CP or FLU ES patch.

Following removal of the well insert each sample was imaged using light microscopy at 4 defined points, giving the reference time point of 0 h. Samples were then returned to the incubator and at predefined timepoints of 2, 4, 6, 8, 12-, 24-, 30- and 36-h samples were removed and imaged to allow for continuous monitoring of the wound closure. At each time-point the patch was removed from the well using sterile forceps and retained for replacement after imaging. Samples were imaged at the same location as T0 using marked reference points, the patches were replaced in the wells and samples returned to the incubator.

Following image collection analysis was conducted using Image-Pro v11 software with the wound healing protocol in the cell biology plus package. All groups were run in triplicate with 4 analysis regions on each sample.

### Statistical analysis

Data is expressed as the mean ± standard deviation calculated from *n* = 5 for mechanical analyses, *n* = 3 for drug release studies and *n* = 4 for biological studies. All results were analysed using one-way analysis of variance (ANOVA) accompanied by Turey’s post hoc test using GraphPad Prism 10. Here, asterisks are used to visually represent the statistical significance on graphs where * represents *P* ≤ 0.05, ** represents *P* ≤ 0.01, *** represents *P* ≤ 0.001, and **** represents *P* ≤ 0.0001. Exact *P* values can be found in the Supplementary Information 15.

## Results

### Optimisation of electrospinning parameters

During the optimisation of the electrospinning parameters several variables were considered. The first being the overall concentration of polymer in the electrospinning solution, here concentrations of 5 and 10% (w/v) were assessed, as shown in Supplementary 1 and 2, respectively. With the physical properties of each sample group, which formed distinct fibres summarised in Supplementary 3.

**Fig. 1:**
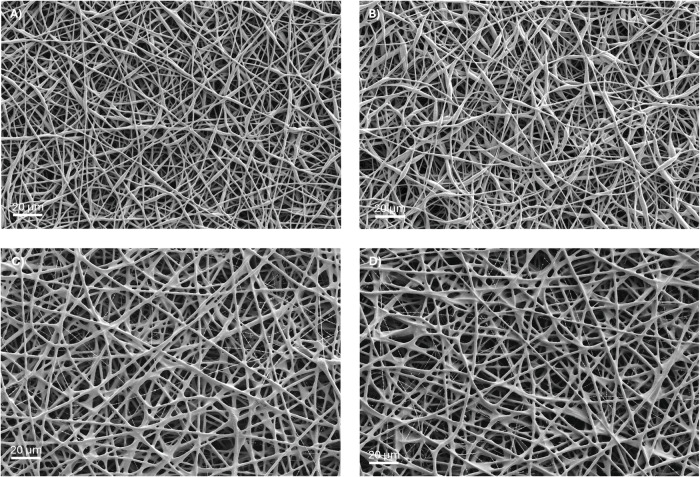
SEM images of electrospun scaffolds, which were chosen for further characterisation based on the initial morphological analysis. **A** 75:25, 10% (w/v), 0.75 mL.hr^−1^, 15 kV at 20 cm (ES1), (**B**) 80:20, 10% (w/v), 0.75 mL.hr^−1^, 20 kV at 20 cm (ES2), (**C**) 75:25, 10% (w/v), 1 mL.hr^−1^, 15 kV at 20 cm (ES3), (**D**) 80:20, 10% (w/v), 1 mL.hr^−1^, 20 kV at 20 cm (ES4)

Following the screening of 3 different blend ratios of MCL:SCL, 90:10, 80:20 and 75:25 at applied voltages of 12 kV, 15 kV and 20 kV 4 candidates for further characterisation were chosen. Following the visual inspection of SEM images these candidates for further characterisation were based on the evidence of discrete fibre formation, with bead-free morphology and homogenous fibre diameter. Given these criteria for acceptable fibre formation none of the 5% (w/v) samples was selected for advancement as the fibres in all groups shown in Supplementary 1 appear melted and fused together, particularly those with the 90:10 MCL:SCL ratio which show little to no porosity in the electrospun structure. The samples electrospun with the 10% (w/v) solutions showed more promising results with both the 80:20 and 75:25 (MCL:SCL) groups allowing the formation of discrete fibres with porous structure. In the 80:20 group a voltage of 20 kV was the only sample group allowing the formation of bead-free fibres. However, the 15 kV voltage proved better for the 75:25 group giving more homogenous fibre diameters than that obtained using the 20 kV voltage as demonstrated in Supplementary 2. Following identification of these parameters a slower flow rate of 0.75 mL.hr^−1^ was investigated to determine if this could improve the homogeneity of the fibres obtained. Figure 1 shows the results from this study, as observed in Fig. 1A, B it appears that the lower flow rate introduces some minor beading in the samples. Given the minor nature of this beading all 4 sample groups shown in Fig. 1 were progressed for further characterisation.

**Fig. 2:**
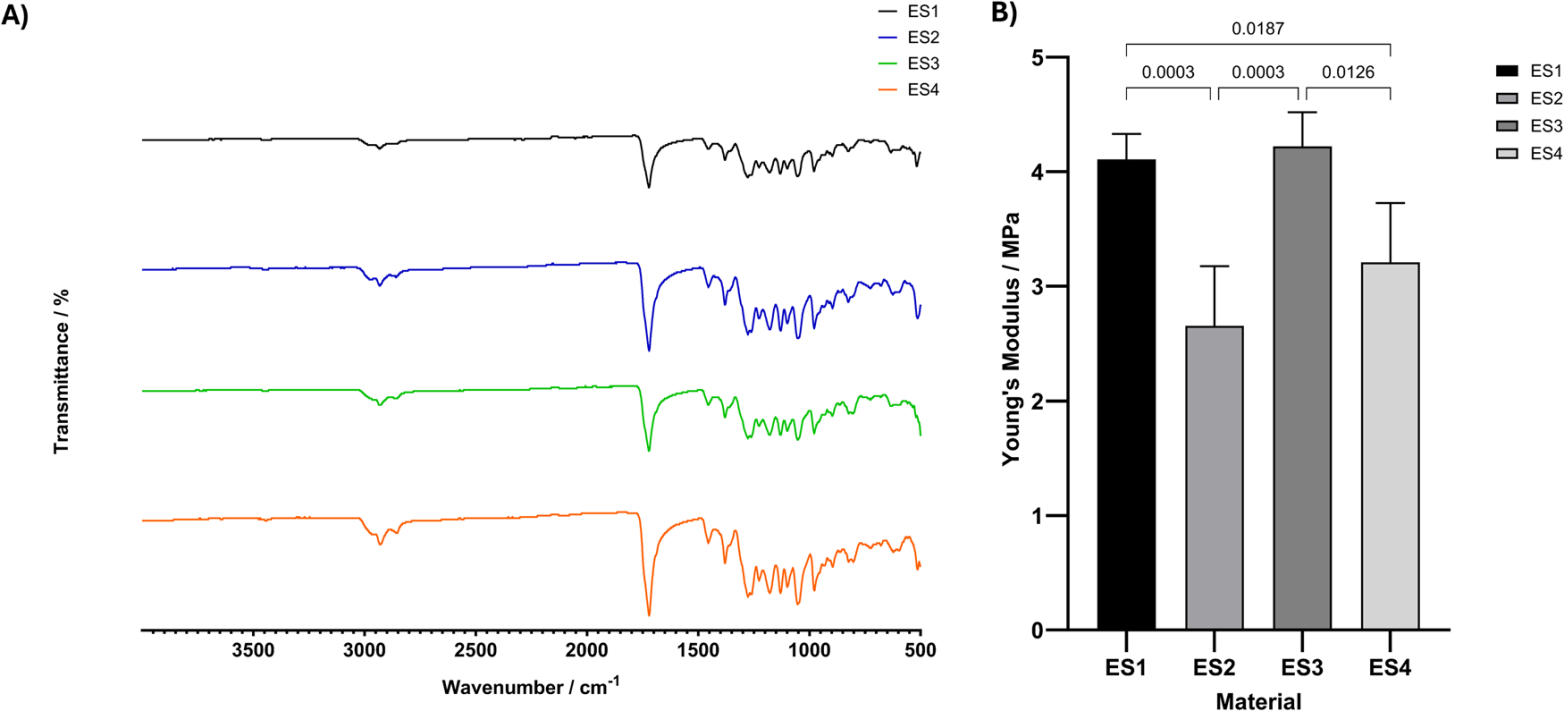
**A** Representative FTIR spectra of MCL:SCL PHA electrospun blends, showing characteristic peaks of each of the individual PHA polymers *n* = 3. **B** Mean Young’s modulus of the electrospun scaffold groups of MCL:SCL blends electrospun fibres tested using a tensile testing method under ambient testing conditions *n* = 5

ES1-ES4 were further characterised using FTIR and DMA for chemical and mechanical analysis, respectively. Figure 2A shows the FTIR spectrum of each electrospun polymer blend, all spectra look similar, showing characteristic peaks of both the MCL and SCL PHAs. The mechanical properties (Fig. 2B) were investigated using a tensile testing method, under ambient conditions, full data shown in Supplementary 4. Results show that the 80:20 blends were in both cases significantly more elastic than their 75:25 counterparts, at dispensing rates of both 0.75 mL.hr^−1^ and 1 mL.hr^−1^. Given the more appropriate mechanical properties of the 80:20 blends for soft tissue, wound healing applications this formulation was chosen for further investigation. There was no significant change in the mechanical properties of the electrospun 80:20 blend at 1 mL.hr^−1^ compared to 0.75 mL.hr^−1^ and therefore the 1 mL.hr^−1^ flow rate was chosen as when scale up is necessary this faster flow rate is advantageous in the production line and makes the process more efficient and cost effective.

### Characterisation of drug loading for wound care applications

#### Clobetasol propionate-loaded fibres

**Fig. 3:**
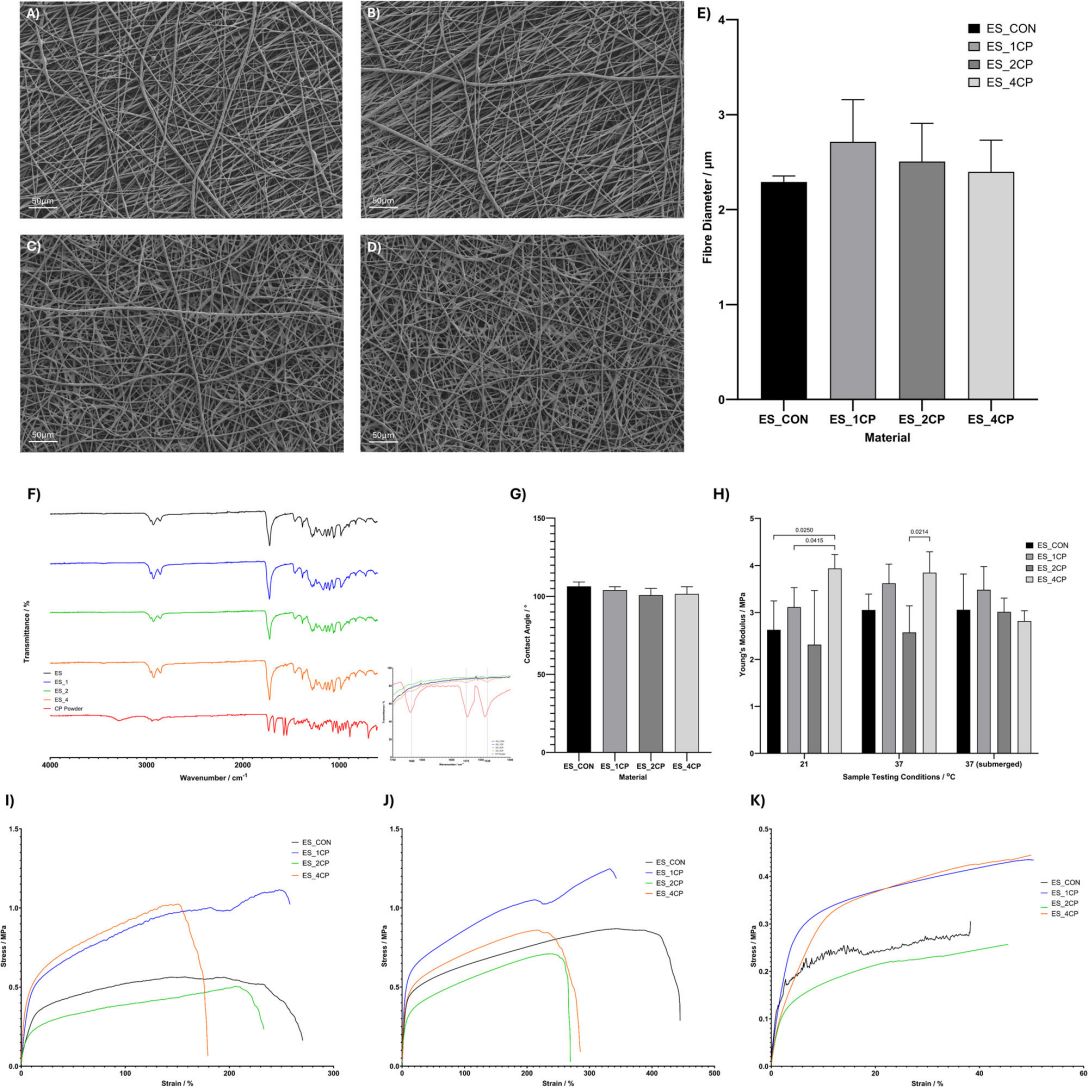
**A**–**D** SEM images showing electrospun fibres manufactured using 10% (w/v) 80:20 MCL:SCL at a flow rate of 1 ml/hr, voltage of 20 kV on a static collector. **A** Native scaffold, (**B**) Containing 1% CP (ES_1CP), (**C**) Containing 2% CP (ES_2CP), (**D**) Containing 4% CP (ES_4CP), (**E**) Average fibre diameters calculated using Image-Pro. **F** FTIR spectra showing for native and CP-loaded fibres and control CP powder. **G** Contact angle measurements indicating the surface wettability of control and drug-loaded samples. **H** Statistical analysis of mechanical properties of control and CP-loaded fibres (**I**–**K**) Stress-strain curves for electrospun MCL:SCL PHA blends at a ratio of 80:20 containing 0–4% CP under a range of testing conditions, (**I**) ambient conditions, (**J**) 37 °C and (**K**) under submersion at 37 °C

The loading of a corticosteroid was investigated due to reports of enhanced wound healing in these patient groups, which display abnormal inflammation during tissue repair processes [53]. The chosen concentrations for investigation were 1, 2 and 4% w/w of the polymer in the electrospinning solution. Figure 3 shows the SEM images of fibres with increasing concentration of CP (A–D) and the calculated mean fibre diameters at increasing concentrations of CP incorporation (E). The final mean diameters of the samples were not significantly changed at any concentration of CP loading, suggesting that the CP is well incorporated into the polymer fibres.

FTIR was used to identify the presence of CP in the electrospun fibre structure (Fig. 3F) and peak assignments investigated in Table 5, given the low concentration of drug compared to polymer and the overlapping of many of the bonds in the polymer and drug chemical structure it is difficult to see any obvious changes in the spectrum following the addition of CP. However, upon close inspection there are some additional small peaks visible in the 4% CP-loaded fibres. These are seen at 1668 cm^−1^, 1575 cm^−1^ and 1539 cm^−1^ which may correspond to the C-F bonding present in the CP chemical structure [54].

Given the necessity for the wound dressings to maintain structural integrity throughout the application time, the mechanical properties of the drug-loaded samples were investigated. Three different conditions were analysed to determine any changes in the mechanical properties which may occur in conditions closer to the in vivo scenario. The Young’s modulus was calculated based on the initial linear elastic region of stress-strain curves, shown in Fig. 3I–K, with the Young’s modulus being significantly increased in the 4% CP fibres under both dry testing conditions. However, under submersion testing conditions this difference in elasticity became statistically negligible as summarised in Fig. 3H.

Thermal properties of the CP-loaded electrospun fibres were investigated using DSC. As seen in Supplementary 5 and demonstrated by the data in Table 2, all samples exhibited two endothermic peaks: the first peak at around 48 °C corresponding to the melting of the MCL-PHA, and the second peak at around 170 °C corresponding to the melting of the SCL-PHA. Additionally, upon the second heating cycle a cold crystallisation peak is observed at around 5 °C for the neat polymer electrospun fibres and between 8 and 9 °C for the samples containing the CP.

**Table 2:** Summarising the thermal events detected using DSC for the native and CP-loaded electrospun fibres during a heat-cool-heat cycle values are a mean of 3 replicate samples and stated as mean ± standard deviation

Heating cycle	Thermal event	ES_CON (°C)	ES_1CP (°C)	ES_2CP (°C)	ES_4CP (°C)
1st heating	Glass transition	N/A	N/A	N/A	N/A
	MCL melting	47.67 ± 0.41	48.17 ± 0.19	48.67 ± 0.33	48.21 ± 0.12
	SCL melting	178.10 ± 0.49	177.51 ± 2.30	176.31 ± 4.00	177.41 ± 1.94
Cooling	Crystallisation	92.06 ± 5.38	96.17 ± 6.92	94.57 ± 4.62	90.14 ± 3.08
2nd heating	Cold crystallisation	6.09 ± 0.27	7.84 ± 0.47	8.03 ± 0.29	8.45 ± 0.17
	Glass transition	N/A	N/A	N/A	N/A
	MCL melting	39.48 ± 1.08	40.72 ± 0.43	39.05 ± 1.32	40.90 ± 0.27
	SCL melting	170.92 ± 1.05	172.47 ± 0.73	171 ± 1.86	171.35 ± 0.18

#### Fluconazole-loaded fibres

**Fig. 4:**
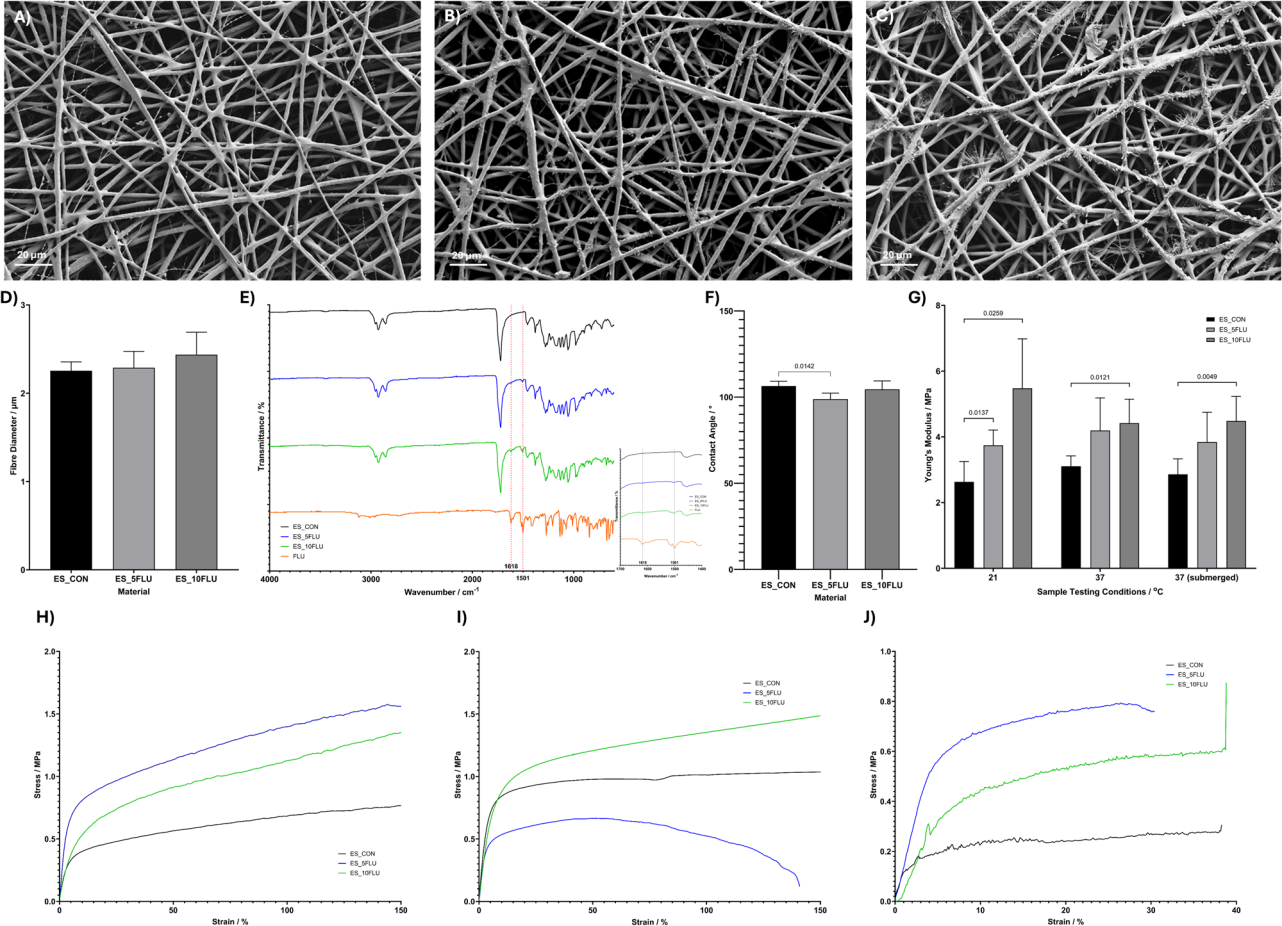
SEM images showing electrospun fibres manufactured using 10% (w/v) 80:20 MCL:SCL at a flow rate of 1 mL.hr^−1^, voltage of 20 kV on a static collector. **A** Native scaffold (ES_CON), (**B**) Containing 5% FLU (ES_5FLU), (**C**) Containing 10% FLU (ES_10FLU) and (**D**) Average fibre diameters calculated using Image-Pro. **E** FTIR spectra showing for native and CP-loaded fibres and control FLU powder. **F** Contact angle measurements indicating the surface wettability of control and drug-loaded samples. **G** Statistical analysis of mechanical properties of control and FLU-loaded fibres. Stress-strain curves for electrospun MCL:SCL PHA blends at a ratio of 80:20 containing 0–10% FLU under a range of testing conditions (**H**) ambient conditions, (**I**) 37 °C and (**J**) under submersion at 37 °C

Following prolonged treatment with corticosteroids many patients experience development of secondary fungal infections, which are commonly treated using FLU. We have investigated the optimisation of loading of FLU in the MCL:SCL fibres for treatment, either following or simultaneously, to treatment with CP. SEM images in Fig. 4A–C show the morphology and homogenous nature of all fibre groups, pre and post FLU loading. Figure 4D shows that there is no significant change in the fibre diameters with increasing concentrations of FLU incorporation.

FTIR was used to identify the presence of FLU in the electrospun MCL:SCL PHA fibres. Figure 4E shows the spectra with comparison to the pure FLU drug. Peaks at 1501 cm^−1^ and 1618 cm^−1^ correspond to peaks seen within the spectra for the pure FLU. The peak seen at 1501 cm^−1^ corresponds to that of the C=N stretching within the triazole found in the chemical structure of FLU. At 1618 cm^−1^ the peak corresponds with the C=C stretching within the aromatic ring seen in the FLU chemical structure [55]. These peaks are not visible in the ES_CON spectrum, and their magnitude appears to increase with increasing concentration of FLU within the scaffold structure. Peak assignments for the overall spectra are investigated in Table 5.

As with the CP-loaded fibres the mechanical properties of FLU-loaded samples were analysed at 3 different experimental conditions under tensile stress. The raw data, stress-strain curves, are shown in Fig. 4H–J. At ambient conditions there was a slightly significant increase both the ES_5FLU and ES_10FLU, this significant change remained at the other testing conditions for the 10% FLU samples but was negated at 37 °C and at the 37 °C submerged conditions for the 5% FLU samples (Fig. 4G).

Thermal properties of the FLU-loaded electrospun fibres were investigated using DSC, the DSC curves are shown in Supplementary 6 with the data summarised in Table 3. Similarly to the CP-loaded fibres, in all samples two distinct endothermic peaks are visible during the 1st heating cycle, the first at around 47 °C corresponding to the melting of the MCL-PHA, and the second again at between 170 °C and 180 °C corresponding to the SCL-PHA melting. In the ES_10FLU sample an additional melting peak is seen at 137 °C which matches the melting temperature of FLU (Supplementary 7), confirming the incorporation of FLU within the fibre structure. With increasing FLU concentration, the crystallisation temperature fluctuated. The crystallisation temperature for the control sample was 95.54 °C and 93.54 °C for ES_5FLU and greatly decreased to 64.7 °C for the ES_10FLU sample. Additionally, the cold crystallisation temperatures fluctuated as the FLU incorporation changed, the cold crystallisation for the ES_CON group was 5.96 °C, with 5% FLU addition was slightly increased to 9.17 °C and further greatly increased to 64.60 °C with 10% FLU incorporation.

**Table 3:** Summarising the thermal events detected using DSC for the neat polymer and FLU-loaded electrospun fibres during a heat-cool-heat cycle values are a mean of 3 replicate samples and stated as mean ± standard deviation

Heating cycle	Thermal event	ES_CON (°C)	ES_5FLU (°C)	ES_10FLU (°C)
1st heating	Glass transition	N/A	N/A	N/A
	MCL melting	47.67 ± 0.41	48.29 ± 0.13	48.48 ± 0.67
	SCL melting	178.10 ± 0.49	175.11 ± 0.40	169.72 ± 1.55
	FLU melting	N/A	N/A	133.37 ± 0.89
Cooling	Crystallisation	92.06 ± 5.38	96.93 ± 6.67	64.13 ± 0.08
2nd heating	Cold crystallisation	6.09 ± 0.27	9.37 ± 0.48	62.71 ± 2.57
	Glass transition	N/A	N/A	N/A
	MCL melting	39.48 ± 1.08	40.55 ± 0.38	40.47 ± 0.65
	SCL melting	170.92 ± 1.05	169.39 ± 1.82	163.50 ± 2.07

### Drug release study

#### Clobetasol propionate-loaded fibres

**Fig. 5:**
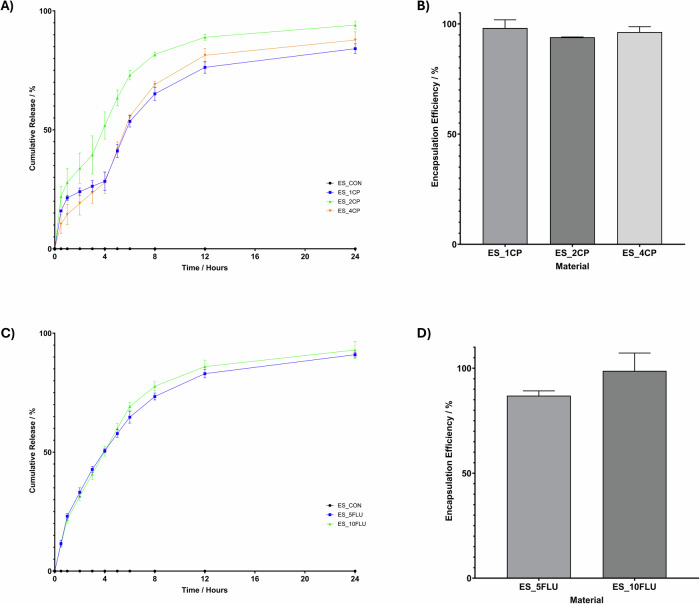
**A** CP release from electrospun MCL:SCL 80:20 scaffolds containing 0–4% CP for 12 h incubation at 37 °C. **B** Loading efficiency of CP in electrospun MCL:SCL PHA electrospun scaffolds. **C** FLU release from electrospun MCL:SCL 80:20 scaffolds containing 0–10% FLU for 24 hours incubation at 37 °C. **D** Loading efficiency of FLU in electrospun MCL:SCL PHA electrospun scaffolds

The drug release profiles, and loading efficiency of CP-loaded fibres are shown in Fig. 5A, B with Table 4 providing supporting data on the loading efficiency study, all samples demonstrated an initial burst release in the first 30 min of incubation. The 2% CP samples released 25.56% of the total drug during this burst release whilst the CP release from the 1% and 4% samples at this time point was much lower at 14.55% and 11.70% respectively, thus demonstrating a much lower burst effect. At 12 h the overall release from the 2% and 4% samples was close to 100%, at 96.57% and 92.31%, respectively. This rate of release is appropriate for wound care applications where dressings are routinely changed every 12–24 h. The release rate is steady over the 12 h, with no severe tailing off, suggesting that the continuous dosing and sustained release over this period should remain above a level which is effective for the treatment of wounds. Loading efficiency of the CP scaffolds was calculated as between 93.9 and 98.11%, whilst this is close to 100% there are several reasons that 100% loading is not achieved. It is noted that the following factors can contribute to drug loss during the electrospinning process, partial drug degradation or volatilization due to the high voltage and solvent evaporation, drug precipitation or phase separation caused by solubility limitations in the polymer solution, and drug loss during collection or post-processing steps such as washing or drying. The maximum percentage saturation of the release media compared to maximum solubility of CP in PBS at 37 °C was 23.89% at 5 h as described in Supplementary 11.

**Table 4:** Detailed raw data showing the theoretical and experimentally determined masses of drug in the electrospun samples

Sample group	Mean sample mass/mg	Theoretical mass of drug/µg	Mean area under HPLC curve/a.u.	Experimental mass of drug in sample/µg
ES_1CP	7.6	76	270.77	74.56
ES_2CP	8.1	162	593.42	152.17
ES_4CP	8.3	332	1246.90	319.73
ES_5FLU	9.2	460	75.64	399.45
ES_10FLU	9.2	920	171.98	908.09

#### Fluconazole-loaded fibres

Figure 5C, D shows the percentage FLU released from electrospun fibres as a function of time and the FLU loading efficiency, respectively, with Table 4 providing supporting data on the loading efficiency study. The prepared samples can provide sustained release of FLU for at least 24 h. Additionally, all release profiles show similar trends. Therefore, as expected, the total amount of FLU released after 24 h increased with drug loading. At 12 h the FLU release from samples with 5% and 10% FLU loading was 83.0% and 85.9%, respectively. Given that these patches are proposed for infection control when applied in a wound care and healing scenario, this timescale of release seems within boundaries as the routine wound care requires cleaning and dressing changes every 12–24 h. Again, 100% loading efficiency was not achieved for the FLU-loaded fibres, this is attributed to the same causes as for the CP-loaded fibres. However, the observed average loading of 86.92–98.72% are acceptable for this manufacturing technique. The maximum percentage saturation of the release media compared to maximum solubility of FLU in PBS at 37 °C was 4.39% at 0.5 h as described in Supplementary 12.

### Biological characterisation of the drug-loaded fibres

#### Clobetasol propionate-loaded fibres

**Fig. 6:**
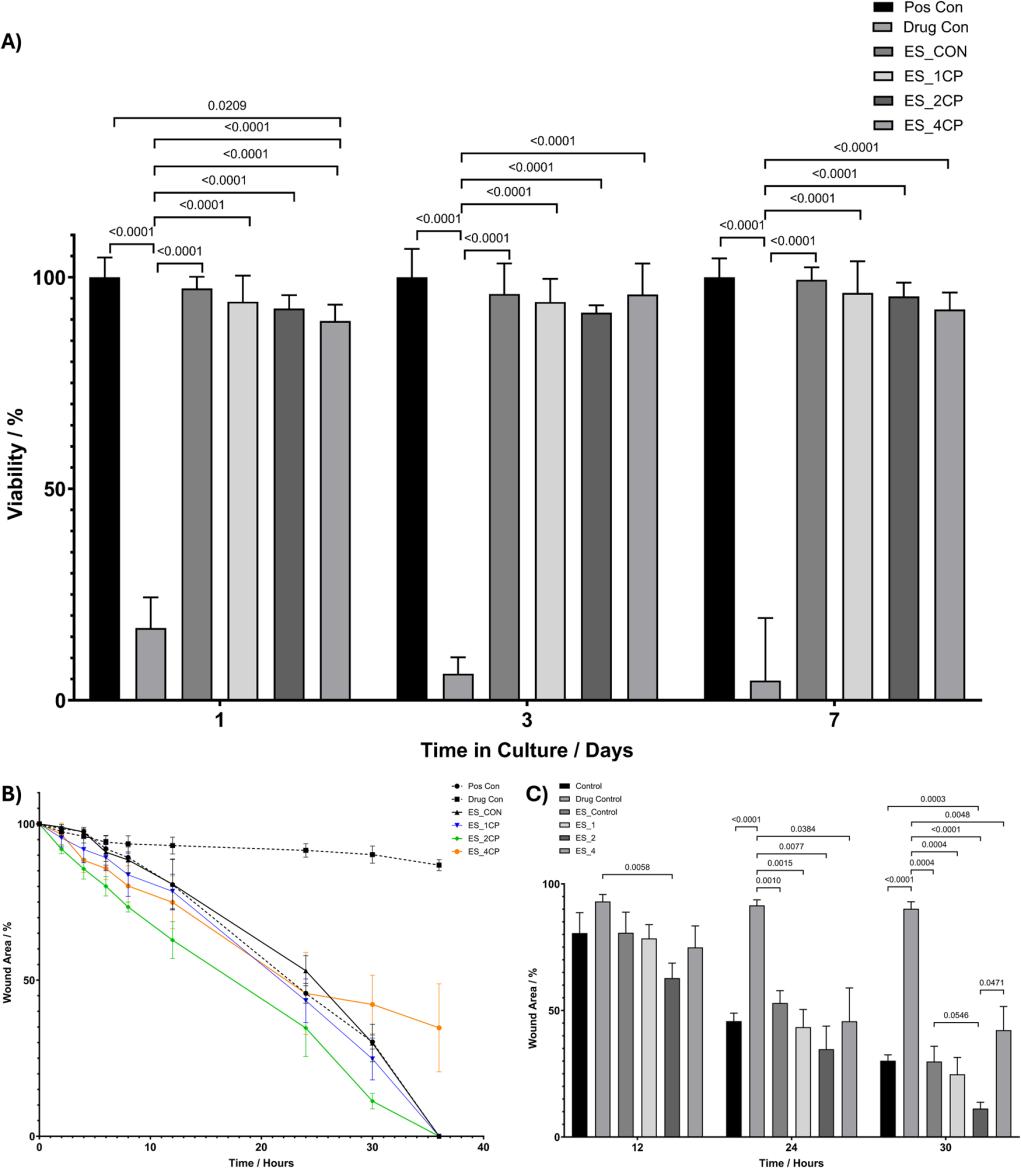
**A** MOE-1a cell viability measured using the MTT assay at day 1, 3 and 7 after addition of the MCL:SCL electrospun scaffolds containing 1, 2 and 4% CP. **B** Percentage wound gap closure of MOE1a cells over 36 h incubation, following addition of electrospun patches containing 0–4% CP to experimental wells. **C** Data analysis showing the differences in wound closure between 12 and 30 h after CP ES patch application to MOE1a in vitro wound healing experiment

To assess the cytocompatibility of the drug-loaded fibres on MOE-1a cells the MTT assay was used (Fig. 6A). The free CP caused significant reduction in the MOE-1a viability at all timepoints. The loading of the CP in the MCL:SCL electrospun fibres prevented the toxic effect of this dosage of CP as very little can be seen in terms of significant changes in the viability of MOE-1a cells, especially in the ES_1CP and ES_2CP samples, up to 7 days of culture. At day 1, there was a slight reduction in the cell viability for the 4% CP samples, suggesting that the initial release from these samples exceeds that which the MOE-1a cells can withstand.

Given the proposed application of the treatment of ulcerative wounds, a wound healing assay was investigated to determine an optimised loading of CP for wound healing applications. The results shown in Fig. 6B show the reduction in wound area over time, using the wound area at T0 as the measure of 100% wound area. The graph shows a similar trend between the control samples and the electrospun fibres containing 1 and 2% CP, suggesting that there is no impedance of the wound gap closure by the presence of the drug-loaded patches. The fibres with higher CP loading (4%) also followed this trend during the initial time-points, however after 24-h it was evident that the rate of wound healing was reduced, and no further significant gap closure was observed up to the final 36-hour time-point. The phase contrast images shown in Supplementary 13 show the samples at 30 h, it can be seen that the MOE-1a cells in the ES_4CP samples appear rounded suggesting that they may be detaching, and many floating cells could be seen in the culture media on these samples. A statistical analysis of the wound area at 12, 24 and 30 h showed that the inclusion of the ES_CON, ES_1CP and ES_2CP patches did not hinder the wound healing process in comparison to the positive control. In fact, at 30 h, it appeared that the remaining wound area for the ES_2CP samples was significantly less than that of the positive controls. This is unexpected as the mechanism of action of CP involves the regulation of inflammatory responses, and one limitation of our in vitro wound healing model was lack of inflammatory stimulation or presence of immune cells. Therefore, we propose that this perceived increased rate of wound closure is due to the prolonged low dosage of CP causing a non-specific proliferative effect. This is a noted in the literature relating to CP for other cell lines and it is possible that the MOE1a cells may also exhibit this increased proliferation in the presence of continual low dose of CP [56, 57]. In all cases the electrospun patches loaded with CP prevented the stalled, non-healing and cell detachment that was observed for the free drug group. Samples incubated with the free drug did not exhibit any gap closure and as shown in Supplementary 13, these samples showed cells with abnormal morphology compared to the usually polygonal epithelial-like morphology of the MOE-1a cells.

#### Fluconazole-loaded fibres

Analysis of cytocompatibility of the FLU-loaded fibres was conducted using the MTT assay and MOE-1a cells. A group containing free FLU at a concentration of 3% w/v in complete growth media was included as the drug control. At day 1 the viability with the control FLU was significantly increased compared to all other groups, however ay the later timepoints of 3 and 7 days the FLU control group appeared to have reduced viability compared to all other groups. This suggests that at this concentration the FLU alone has cytotoxic properties. However, there was no decrease in viability recorded with any of the electrospun samples compared to the positive control group. These results are positive as no cytotoxicity is associated with the electrospun samples; therefore, they can successfully be incorporated into dressings for wound healing applications.

**Fig. 7:**
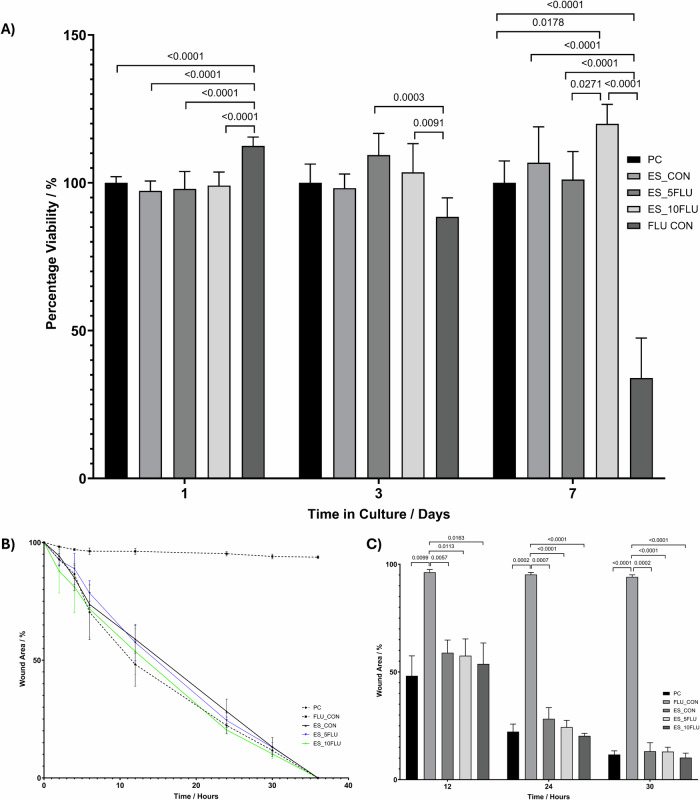
**A** MOE-1a cell viability measured using the MTT assay at day 1, 3 and 7 after addition of the MCL:SCL electrospun scaffolds containing 5 and 10% FLU. **B** Percentage wound gap closure of MOE1a cells over 36 h incubation, following addition of electrospun patches containing 0–10% FLU to experimental wells. **C** Data analysis showing the differences in wound closure between 12 and 30 h after FLU ES patch application to MOE1a in vitro wound healing experiment

A wound healing assay was conducted, also using the MOE-1a cells, to ensure no inhibition of the wound healing process is caused by the electrospun samples with FLU incorporated. Results shown in Fig. 7B show that all experimental samples followed a similar trend to that of the positive control during closure of the wound gap. The drug control samples did not heal and as seen in Supplementary 14 the wound gaps remain at the 30-h time point. Data analysis, summarised in Fig. 7C, of the wound area showed that the experimental samples showed no significant change in comparison to the positive controls. The electrospun samples therefore are not impeding the wound gap closure whilst the free drug prevented wound healing. In line with the viability results presented in Fig. 7A it appears that the free drug at this concentration causes a cytotoxic effect as images included in Supplementary 14 show rounding and detachment of cells at the 30-hour time point for this sample group.

## Discussion

Visual observation of the SEM images (Supplementary 1) quickly revealed that solutions which contained high ratio of MCL-PHA could not be successfully electrospun to obtain discrete individual fibres at a solution concentration of 5% (w/v). At all voltages investigated the fibres appeared fused with areas of solid polymer among the successful fibres in some cases. With increasing concentration of SCL-PHA the electrospinning was somewhat more successful. In most cases the 90:10 samples formed fused films with no evidence of fibres, whilst some fibre structures were seen in the 80:20 and 75:25 samples at the polymer concentration of 5% (w/v). Previously, Li et al. showed that the lowest concentration of SCL-PHA required for the successful electrospinning to obtain discrete fibres with diameters fitting a normal distribution profile was 65% [35]. In this study, they show the best fibre properties are obtained using a 25:75 MCL:SCL ratio however under these conditions the material is too stiff for the current proposed application. The lack of success in electrospinning high ratio MCL solutions at 5% (w/v) prompted the investigation into electrospinning at a higher overall polymer concentration to achieve a higher viscosity and increase molecular entanglements to encourage the formation of discrete, bead-free fibres [58]. The SEM images presented in Supplementary 2 summarise the physical properties of the electrospun fibres obtained during this investigation. It is seen that the 90:10 solution is unsuccessful at forming any kind of fibres, however, both the 80:20 and 75:25 solutions show some success at the 10% (w/v) concentration. At the 75:25 ratio all samples contain discrete fibres with uniform morphology. The 80:20 samples exhibited considerable beading at lower voltages, however this beading was eliminated by using a higher voltage for electrospinning. The 75:25 and 80:20 blends were further characterised at flow rates of 0.75 mL.hr^−1^ and 1 mL.hr^−1^ for selection of the most appropriate manufacturing conditions for the proposed application. Figure 1 shows that all 4 of these formulations result in smooth, bead-free fibres with uniform fibre diameters.

Using FTIR the electrospun fibres were analysed for their chemical composition. As seen in Fig. 2A the most prominent band in all FTIR spectra was located at around 1720 cm^−1^ corresponding with the ester carbonyl (C=O) absorption band, which is the main functional group of PHAs. Previous groups have reported a shift in this band with different concentrations of MCL-PHA, which, due to its amorphous nature shifts this peak towards 1730 cm^−1^ [59]. However, it is likely that the difference in the blend ratios of MCL:SCL PHAs in the current study was not high enough to give rise to such observations.

Given one of the key questions in this study was the potential to enhance the elastic properties of electrospun PHA blends the mechanical properties of the fibres were tested (Fig. 2B). Results of the tensile test showed that the 80:20 blends had significantly lower Young’s modulus at flow rates of both 0.75 mL.hr^−1^ and 1 mL.hr^−1^ when compared to the 75:25 equivalents, suggesting that the samples with higher MCL-PHA ratio have increased elasticity. Values of Young’s modulus for the electrospun samples ranged from 2.65 to 4.22 MPa. Soft tissues have highly anisotropic properties, with mechanical properties being dependent on tissue location, direction of loading and age of tissue [60]. Reported values of Young’s modulus for skin lie anywhere between 0.3 and 20 MPa [61], whilst values for oral mucosal tissue range from 0.91 to 11.12 MPa [62], when tested under tensile conditions at quasistatic speeds such as those used in the current study. Therefore, the materials produced via electrospinning in this study appear to have appropriate elasticity for wound healing applications as they lie within these ranges. The successful electrospinning of MCL:SCL blend solutions with high MCL content has effectively reduced the Young’s modulus of the samples compared to previous studies in which ratios were 50:50 MCL:SCL or majority SCL PHA, which resulted in Young’s modulus values of 34.46 MPa [35]. Ching et al. have also described the blending of different PHAs to tune the stiffness of the electrospun scaffolds. Results showed a much lower Young’s modulus than that obtained in our study, within the 300–800 kPa range, even for fibres which were composed of higher SCL content [28]. However, this data was obtained via indentation force analysis, which has previously been shown to give significantly lower Young’s modulus values than measurement via tensile testing techniques. A review study by McKee et al. showed that there are reported increases in Young’s modulus of up to 667 times when measured via tensile methods in comparison to indentation methods [63]. The study by McKee et al. shows the importance of direction and type of loading in mechanical testing and may explain the reason for Ching et al. obtaining such low Young’s modulus values at high SCL-PHA content. Considering the combined results from these two studies, [28, 63], it is likely that the materials produced by Ching et al. would in fact have a much higher modulus than the electrospun blends produced here, if tested under tensile conditions similar to those used in our study. Elsewhere other groups have described the use of co-polymers of MCL and SCL PHAs to enable the optimisation of mechanical properties giving significantly reduced Young’s modulus values at molar percentages of MCL PHA higher than 38% [32]. The potential to tailor the mechanical properties of electrospun SCL-PHAs using deep eutectic solvents has been evaluated, and whilst a significant reduction in Young’s modulus of 76% could be achieved using acetyl tributyl citrate, the modulus value was still high at 60 MPa, compared to the values obtained in our study via blending with MCL-PHAs [64]. Volova et al. have investigated the effect of fibre diameter on elasticity of electrospun PHAs, where increasing the fibre diameter of SCL-PHAs allowed significant change in Young’s modulus from 1.16 to 0.3 GPa [32]. This approach showed promise in tailoring mechanical properties for some applications, but these values still describe a material which is far too stiff for soft tissue applications described in our study.

Given the results obtained in in Figs. 1 and 2 the 80:20 MCL:SCL polymer blend was chosen for further production of the drug-loaded samples as this electrospinning solution gave the best elastic properties for the application of wound healing. Since there was no significant difference in the Young’s modulus at flow rates of 0.75 mL.hr^−1^ or 1 mL.hr^−1^ the flow rate selected was 1 mL.hr^−1^, as for industrial scaleup this flow rate would be much more time efficient to produce thick samples for wound dressings. These optimised electrospinning parameters were used in the subsequent experiments describing the optimisation of fibres for delivery of clobetasol and fluconazole.

Drug-loaded samples (CP and FLU) were initially visually examined using SEM for assessment of the physical properties. As seen in Figs. 3A–D and 4A–C the incorporation of the drugs in the electrospinning solutions caused some changes in the morphology of the fibres without significantly effecting the fibre diameters. In the ES_4CP (Fig. 3D) some precipitation of the drug is seen within the fibre structures. This is likely as at this concentration of CP the electrospinning solution is approaching a saturated state as the solubility in chloroform is reported as 50 mg.mL^−1^. The solubility of FLU in chloroform is poor, therefore at both concentrations of 5% and 10% we see evidence of drug separation in the fibres, with these structures becoming more frequent as the drug concentration increases. Whilst in the past groups have worked to fully encapsulate the drug within the fibre structure this is usually to ensure that drug release is sustained over long periods for implantable materials [65, 66], here we aim to produce a wound dressing which will be in place for a maximum of 12–24 h before removal/replacement, meaning the exposure of drug at the fibre surfaces may be advantageous in the drug release process to ensure maximal delivery within this timeframe.

The FTIR analyses in Figs. 3F and 4E, show the potential evidence of drug incorporation in the fibre structure. The spectra for CP (Fig. 3F) show the increases in intensity for the ES_4CP with small peaks seen at 1575 cm^−1^ and 1658 cm^−1^ which correspond to peaks seen in the pure CP spectrum. These wavenumbers correspond to those expected for C-F bonding present in the CP chemical structure [54]. These peaks were only seen at the highest concentration of CP incorporation in the fibres; this may be due to the exposure of the CP at the fibre surfaces in the 4% samples as highlighted in Fig. 3D. There is significant overlapping of many of the peaks for CP with the peaks associated with the polymer itself which may explain why at lower concentrations no peaks corresponding to the CP can be detected. The FLU-loaded fibres show clearer evidence of the drug incorporation with distinct peaks, which appear to increase in intensity as the drug concentration increases from 5% to 10%, seen at 1501 cm^−1^ and 1618 cm^−1^ which align with peak seen in the pure FLU sample. Within the FLU chemical structure there is a C=N bond contained within a triazole, which is evidenced by the peak seen at 1501 cm^−1^ [55]. Additionally, in the 10% sample a small peak is observed at 1618 cm^−1^ which is indicative of the C=C bond within the aromatic ring in the FLU chemical structure [55, 67]. Despite the addition of these small peaks indicating the incorporation of CP and FLU within the polymer fibres there is no shifting of any of the characteristic polymer peaks, detailed in Table 5. This suggests that the incorporation of the polymer is not via any chemical interaction between the polymer and the drug, instead the drug molecules are simply physically entrapped within the polymer matrix.

**Table 5:** Peak assignment table for the FTIR spectra of MCL-PHA, SCL-PHA and drug (CP&FLU) incorporated electrospun scaffolds

Wavenumber/cm^−1^	Bond attribution	Material attribution
2950	-CH_3_	MCL- & SCL-PHA
2924	-CH_2_-CH_2_-	MCL- & SCL-PHA
2854	-CH_2_-CH_3_-	MCL- & SCL-PHA
1722	-C=O	MCL- & SCL-PHA
1658	C-F	CP
1618	Aromatic C=C	FLU
1575	C-F	CP
1501	C=N	FLU
1462	-C-H bend	MCL- & SCL-PHA
1436	-C-H bend	MCL-PHA
1378	-C-H bend	MCL- & SCL-PHA
1316	O-H bend	MCL- & SCL-PHA
1376	-CH_3_	SCL-PHA
1280	-CH_3_ bend -CH_2_ wagging	SCL-PHA
1260	-C-O-C-	SCL-PHA
1224	Conformational band	SCL-PHA
1178	-C-O stretch	SCL-PHA
1126	-C-O stretch	SCL-PHA
1160	-C-O stretch	MCL-PHA
1128	-C-O stretch	MCL- & SCL-PHA
1096	-C-O stretch	MCL- & SCL-PHA
1044	C-O stretching	MCL- & SCL-PHA
976	C=C bend	SCL-PHA
894	C=C bend	SCL-PHA
826	C=C bend	SCL-PHA
724	C-C	MCL-PHA

Data interpretation is made from the following refs. [101–104].

The mechanical properties were investigated following the addition of CP and FLU to the electrospinning solution, as it is reported that the interactions between the drug molecules and polymer matrix can cause significant changes in the mechanical properties of electrospun membranes. To date, most reports of electrospun PHA-based drug delivery materials have been based on the SCL P(3HB) polymers, resulting in brittle scaffolds with low impact resistance [68–70]. When drugs are loaded into electrospun fibres they can often become stiffer due to these interactions causing increased solution viscosity leading to less flexible materials with higher Young’s modulus [46, 71]. Results summarised in Figs. 3H and 4G demonstrated this effect with the inclusion of both CP and FLU in the fibre structure causing significant increases in the Young’s modulus when tested under ambient conditions. This increase in stiffness appears to align with where drug-saturated electrospinning solutions were used with the ES_4CP, ES_5FLU and ES_10FLU samples. In this scenario where the drug is not fully dissolved in the electrospinning solution, we can see via SEM images (Figs. 3 and 4) that the drug molecules become like a filler material within the fibres. These rigid fillers increase the overall stiffness of the drug-loaded fibres. Additionally, they may introduce defects into the fibre structure thus increasing the potential weakness points within the material, this phenomenon has been observed frequently throughout the literature when considering the introduction of filler type materials into polymeric electrospun fibres [72, 73]. However, the mean Young’s modulus remained below 10 MPa in all cases and given the ranges reported for the stiffness of dermal and oral mucosal tissue these values all seem appropriate for the intended applications [61, 62]. The values for Young’s modulus obtained in our study lie towards the lower end of the previously quoted range for the oral mucosa of 0.91–11.12 MPa. Given that the majority of persistent oral inflammatory sores are located on the buccal mucosa [74, 75], which presents with lower Young’s modulus, as opposed to gingival or hard palate the mechanical properties of the obtained scaffolds in this study are appropriate for the application [76]. A previous study investigating the tensile properties of different gingival and buccal regions of porcine soft tissue showed Young’s modulus of between 2.48 ± 0.37 and 5.74 ± 1.15 MPa for regions associated with the buccal tissue, which correlates well with the values obtained in our study [77]. Whilst this is not an ideal comparison due to the use of porcine tissue in the study by Goktas et al., the oral mucosa of pig has been previously proven to be one of the most comparable to that of human tissue for use in research purposes [78]. Elsewhere, a group have provided a comparison of the Young’s modulus of human gingival, hard palate and buccal mucosal tissue. Whilst the values for the buccal mucosa in this study were slightly higher at 8.33 ± 5.78 MPa, the high standard deviation means that this is not significantly different from the values obtained for the Young’s modulus of the samples prepared in this study [60]. Interestingly, under submerged conditions this effect is minimised with there no longer being a significant difference between the control samples and the ES_4CP or ES_5FLU. This may be explained due to the interaction of liquid with the polymer-filler interface [79, 80]. This effect has been observed throughout the literature on a range of different polymers, dry vs hydrated fibrinogen electrospun samples demonstrated around a 100 times reduction in the Young’s modulus when tested under hydrated conditions [10]. Goh et al., showed a similar trend when testing polyacrylonitrile electrospun fibres under both dry and wet conditions where the presence of water resulted in a decrease in Young’s modulus from 274 MPa to 24.6 MPa [81]. The difference in the elastic properties during water submersion were not as significant using the materials investigated in our current study, perhaps due to the more crystalline nature of the SCL-PHA not presenting so many amorphous regions for the exposure of polymer chain ends. The tightly packed, ordered structure of crystalline regions restricts the free space for the penetration of solvent molecules, which expand the polymer chains causing the reduced stiffness [82]. However, all samples tested within our study appear to have suitable properties for wound dressing uses, whether that be for application to dermal wounds in drier environment or in a mucosal environment where there is some moisture interference with the dressing material.

DSC thermograms revealed various thermal properties of the scaffolds and components investigated. The data is summarised in Tables 2 and 3 with representative thermograms supplied in the Supplementary 5, 6. The raw drugs, CP and FLU, each demonstrate a single strong endothermic peak at 199.78 °C and 138.17 °C, respectively. The melting temperatures of pure MCL-PHA and SCL-PHA were 52.52 °C and 178.04 °C and following solubilisation and blending in CHCl_3_ followed by electrospinning these melting temperatures did not significantly change. These melting temperatures reveal the comparatively high crystallinity of the SCL-PHA compared to MCL-PHA, this can also be confirmed by the presence of glass-transition, crystallisation and cold-crystallisation peaks seen in the DSC thermogram for the pure SCL polymer, shown in Supplementary 8. All, of the drug-loaded scaffolds showed similar melting points for both the MCL and SCL PHA within a narrow temperature range. For the CP-loaded scaffolds, there was no evidence of the melting peak for the drug, possibly due to the very low concentration of drug in comparison to polymer within the matrix or full solubilisation of the drug in the electrospinning solution may have converted it to an amorphous state [83]. A slight change in the cold crystallisation temperature pre and post CP loading was observed. The CP-loaded samples showed increasing cold crystallisation temperature as the degree of loading increased, this may be owing to the interactions of the drug with the polymer chains. The presence of the drug within the polymer may affect the mobility of the polymer chains thus requiring a higher temperature for recrystallisation. A different effect was seen for the FLU-loaded electrospun fibres, where in the ES_10FLU samples a distinct melting peak was observed for the FLU at 134.11 °C. This was slightly lower than the melting temperature of 138.17 °C obtained for pure FLU powder suggesting that the FLU was, at least partially, in the amorphous form in the blend electrospun fibres [84]. This distinct peak was attributed to low drug-polymer affinity between the two materials confirming that there was no chemical interaction between the two. At the 10% FLU concentration a shift in the crystallisation temperature was observed, there are multiple possible explanations for this. One explanation may be the drug acting as a plasticiser by increasing the free volume and segmental mobility of the polymer chains, however when the DMA results are considered this explanation is rendered unlikely as the modulus for the 10% FLU group was increased over controls. The most likely explanation is that at this concentration the drug is disrupting the polymer chain regularity and hindering nucleation and crystal growth due to irregular packing. Evidence of this is clear as this exotherm enthalpy was decreased from an average of 45.65 J.g^−1^ in control electrospun samples to only 6.41 J.g^−1^ in the ES_10FLU samples. Some evidence of this phenomenon is also observed in the ES_5FLU sample group, whilst the crystallisation temperature is not affected a reduction in the enthalpy is seen, with an average normalised enthalpy of 27.53 J.g^−1^ for this sample group. It appears that the incorporation of small amounts of drug into the MCL:SCL PHA polymer blend electrospinning solutions does not significantly impact the thermal properties of the final electrospun scaffolds with the only major changes detected in the 10% FLU samples.

To predict the therapeutic efficacy of the scaffolds an in vitro drug release study was done on both the CP and FLU-loaded electrospun fibres. The release profile for the CP-loaded scaffolds followed similar trends for all different amounts of drug loading. There was an initial burst release within the first 30 min of incubation and following this the rate of release reduced allowing the delivery to be sustained over 12 h. Interestingly, the burst release of the moderately loaded scaffold, ES_2CP, was greater than that of the 1% or 4% loading for CP. In comparison to the 1% loaded system there is a higher overall drug content in the 2% system, which may be the reason for this increased burst release. A higher initial drug concentration created a steeper concentration gradient between the scaffold and the surrounding environment. This may have accelerated the rate of diffusion at the initial sampling time points. Conversely, looking at a comparison of the 2% and 4% sample groups, this no longer seems to be the case as the 4% samples release less percentage of their overall loading during the initial burst release than the 2% CP scaffolds. This may be for various reasons. Perhaps the higher drug concentration is causing a change to the polymer-drug interaction. If the drug concentration is too high this effect may be causing the drug to bze more deeply and uniformly integrated into the polymer matrix, making it less accessible for a rapid burst release. However, more likely in this case is that there is a saturation effect. During the manufacturing the electrospinning solution was close to the saturation point for the drug and this may cause the drug to aggregate. These aggregates within the system have reduced mobility and may contribute to the lower burst release observed here. The steady release over the 12-h period is encouraging for use in wound dressing applications, which are typically removed and replaced every 12–24 h to allow for wound cleaning. The absence of diminishing rate or plateau of release ensures that an adequate dosage of drug is maintained throughout the dressing application, preventing the potential for antimicrobial resistant strains to develop due to inadequate dosing. Overall, the release of the ES_2CP and ES_4 CP-loaded scaffolds seemed most appropriate as the release at 12 h was close to 100%. Almost complete release in the intended patch application time is desirable as it ensures none of the drug is wasted making the patches much more cost effective when using expensive therapeutic molecules. The FLU release was also maintained over 12 h with 72.51% and 81.78% released by the ES_5FLU and ES_10FLU, respectively, at this time point. This shows an improvement in sustained release profile compared to what was found with PVA electrospun fibres, giving a sustained release of FLU over only 7 h [84]. We hypothesise that this difference in release profile is primarily due to different swelling properties of the polymers used as the base material for scaffolds, PVA presents with more hydrophilic properties than our PHA blend and therefore the increased expansion of the polymer network during swelling can allow for increased diffusion coefficient for drug release [85].

Release modelling was carried out to elucidate the release mechanisms involved in the fibrous scaffolds. The R^2^ values of all groups fitted best the Korsmeyer-Peppas model of drug release, as shown in Supplementary 9, 10, this is unsurprising due to the polymeric composition and cylindrical shape of the fibres, both of these are characteristic features which can lead to this release mechanism being observed [86]. The *n* values of the Korsmeyer Peppas model lay between 0.494 and 0.806 for all sample groups suggesting non-Fickian transport mechanism of drug release [87, 88]. This transport mechanism means the diffusion of drug from the polymer matrix is not solely driven by the concentration gradient but also influenced by other factors such as polymer relaxation, swelling or erosion. Importantly for this therapeutic is that throughout the sustained release period the effective dose is maintained to ensure the risk of AMR development is reduced. An investigation by Maenchantrarath et al. defined the minimum inhibitory concentration for over 32 strains of *C. albicans* to be between 0.25 and 2 µg.mL^−1^ [89]. Elsewhere, the MIC_50_ and MIC_90_ of FLU has been described as much higher across 61 clinical isolates of *C. albicans*, this study found these values to be 0.5 and 32 µg.mL^−1^, respectively [90]. Throughout the drug release study performed for the FLU fibres the minimum release was observed at the latest timepoint and corresponded to 34.93 µg.mL^−1^, which remained above the MIC quoted in literature when considering a range of clinically relevant strains of *C. albicans*. The importance of remaining above the MIC for treatment of infection is paramount not only to the treatment efficacy but to prevent the development of antibiotic resistance, which has been reported to be associate with inadequate antimicrobial therapy for infections [91]. This result suggests that the dosage of FLU throughout a 12-h application period will remain effective. There is much evidence throughout the literature that describes the benefits of sustained drug release in regard to mitigating AMR. Sustained release addresses this via minimising underdosing, enabling targeted local delivery, improving patient compliance, and overall increasing treatment efficacy [92].

In vitro biological characterisation of the fibres was undertaken to assess the cytotoxicity of the membranes on oral epithelial cells. Results summarised in Figs. 6A and 7A show that the control electrospun scaffolds presented no evidence of cytotoxicity when compared to positive controls, which is consistent with reports of the cytocompatibility of PHAs with a variety of cell types [93–95]. The study conducted over a 7-day period showed little effect of the CP-loaded membranes on MOE-1a viability with only a slightly significant decrease in percentage viability to 89.7% for the 4% CP samples at day 1. However, given the standards set by ISO this exceeds the threshold of 80% viability, above which indicates that the material is non-cytotoxic [96]. Most interestingly, the loading of the CP within the fibres appears to reduce the cytotoxic effect of the free drug, for which at all timepoints the viability was significantly reduced in comparison to positive controls. This is likely due to the instant exposure to the full drug dose for the free drug samples compared with the gradual release of drug from the fibres over a 12-h period. However, the control drug dosages were difficult to convert from those found in topical ointments as the wound bed tissue exposure to CP during treatment with ointment is poorly studied in the literature hence the 10 µg.mL^−1^ concentration used as a CP control during studies is an approximation of the 0.01–0.05% doses found in topical ointments. Results which resemble those of the viability study were observed throughout the wound healing study. It is clear from the data in Fig. 6B and Supplementary 13 that the control drug dosage is preventing wound healing, it can be seen in the phase contrast images (Supplementary 13) that at the 30 h timepoint the wound area is completely intact, and the surrounding monolayer of cells appeared damaged with cells presenting with rounded morphology and a degree of cell detachment was evident. Conversely, the samples treated with the CP-loaded membranes appeared to match the wound healing trend of that seen in the positive control with all samples being fully healed at 36 h. From the results presented here using our basic in vitro model it seems that the 2% CP scaffold was most appropriate as the wound area is significantly lower than that seen in the control scaffold. This is an encouraging result as it confirms the drug-loaded scaffold is not impeding the wound closure however following the use of our in vitro monolayer study a more complex system should be used to assess the biological efficacy of the drug-loaded scaffold as the mechanism of action of CP is to suppress the chronic inflammation observed around the wound bed of chronic wounds [97]. This mechanism relies on the suppression of immune cells which are not present in the monolayer culture, therefore, to fully study the potential benefits of the CP-loaded patches a co-culture, tissue engineered mucosa or animal model involving immune stimulation should be used in the future. However, the present results are encouraging in confirming that the control and CP-loaded patches do not cause any inhibition to the closure of a wound gap in oral epithelial cells. The same studies were carried out using the FLU-loaded membranes, whilst this drug is not intended to enhance the wound healing potential of the scaffolds it is important to ensure that the inclusion of drug in the system does not impede the cell viability or wound gap closure. The cell viability study revealed that the electrospun PHAs and those containing FLU did not cause any significant cytotoxicity compared to positive controls. This is an improvement on the results seen by Gunduz et al. where their PMMA/FLU scaffolds caused significant reduction in viability compared to controls [98]. Whilst it appears at least some of this reduction in viability was due to the choice of polymer, it is seen that the PMMA/FLU group in the study still showed even greater decrease in viability at day 1 compared to the native PMMA scaffold. When considering these results alongside our own it appears that the delayed rate of FLU release from our fibres may be preserving the cell viability as within the study by Gunduz et al. almost complete drug release was seen at 3 h compared to the prolonged release up to 24 h in our samples. The doses of both CP and FLU delivered from the electrospun scaffolds are non-cytotoxic under the experimental conditions investigated here whilst the free drug controls show relevant cytotoxicity. As seen in Fig. 7B, C alongside Supplementary 14 the inclusion of FLU in the fibre structure does not influence the wound closure. There is no significant difference in the wound area at 12 and 30 h between the positive control and any of the experimental samples. However, again here the presence of the free drug appears to prevent the healing with the wound gap remaining at above 90% of its original area throughout the study. Again, the phase contrast images (Supplementary 14) reflect this as the 30-h drug control sample shows detachment of cells within the monolayer surrounding the wound area. This is consistent with the results seen in the viability study where the free FLU caused a significant cytotoxic effect after 1 day of incubation (Fig. 7A).

## Conclusions and future directions

Initial studies showed that the electrospinning of high content MCL-PHA blends is possible using 80:20 (w/w) ratio of MCL:SCL polymer, at a polymer concentration of 10% (w/v). The ability to electrospin at this high content of MCL-PHA has greatly enhanced the elastic properties of the materials obtained, compared to previous reports of electrospun PHA blends. Enhanced potential to tailor these mechanical properties offers significant opportunities for these materials to be used in biomedical applications such as wound dressing technology and soft tissue engineering.

Given the proposed application of treatment of chronic wounds, ulcers and associated fungal infections we have investigated the optimisation of the dosing of two appropriate drugs, clobetasol propionate and fluconazole. We conclude that most promising dosing of CP is 2% due to the ability to maintain the mechanical properties within the range required for mucosal and other soft tissues alongside good biological properties. Additionally, this loading appeared to provide the most consistent drug release over a 12-h delivery period. The 10% FLU scaffold is identified as the most appropriate choice for the application, despite slightly reduced elasticity compared to controls this sample group provided drug release closer to 100% within a 12-h period and caused no impedance to the wound gap closure or oral mucosal cell proliferation.

Future studies should aim to combine these two scaffolds in a dual targeting system for the spatial delivery of each drug. This dual targeting system is envisioned as a bilayer patch delivering the CP preferentially towards the mucosal lesion, whereas the FLU is delivered universally around the patch to target secondary fungal infections caused as a result of long-term immunosuppression using corticosteroids such as CP. Additionally, some studies into the muco-penetration of the CP should be investigated to determine the efficiency of the drug delivery via transdermal or transmucosal routes. This may be achieved using ex vivo or in vivo methods followed by HPLC analysis of drug concentration. A major limitation of this study is the lack of an immunocompetent model, which should be used to investigate the efficacy of the CP delivery in vitro, in the future this should be done using an inflammatory model of upregulated inflammatory response using monolayer, 3D cultures or an in vitro tissue model to better mimic the in vivo scenarios of chronic inflammation. Following this, in vivo studies using a chronic inflammatory model should be investigated to provide further insight into the tissue-specific delivery and tissue-level drug exposure of CP and FLU, respectively. Additionally, we plan to incorporate a mucoadhesive coating on the patch, utilising polydopamine-inspired chemistry, as previously reported by our group [93]. Studies show that this type of coating does not significantly affect the mechanical properties of the materials, and our own ongoing preliminary studies (data not shown) support these claims [99, 100].

## Supplementary information

Supplementary information
