# Supplementary material for: Optimisation of electrospinning parameters to successfully obtain high ratios of medium chain length polyhydroxyalkanoate in electrospun fibres with drug loading for wound healing applications
Source: J Mater Sci Mater Med. 2026 Mar 26;37(1):56. doi: 10.1007/s10856-026-07030-5 (PMC13043541; doi:10.1007/s10856-026-07030-5)
Supplement: Supplementary file 1 — Supplementary information [file 10856_2026_7030_MOESM1_ESM.docx]

**Optimisation of Electrospinning Parameters to Successfully Obtain High Ratios of Medium Chain Length Polyhydroxyalkanoate in Electrospun Scaffolds with Drug Loading for Wound Healing Applications.**

Robyn A. Macartney^1^, Annabelle T.R. Fricker^2^, Gusti U.N. Tajalla^2,3^, Andrew M. Smith^4^, Shosei Kishida ^5^, Stefano Fedele^6,7^, Ipsita Roy^2,8^, and Jonathan C. Knowles^1*^

* Corresponding Author

^1^ Division of Biomaterials and Tissue Engineering, University College London (UCL) Eastman Dental Institute, London, United Kingdom

^2^ School of Chemical, Materials & Biological Engineering, Faculty of Engineering, University of Sheffield, Sheffield, United Kingdom

^3^ Materials and Metallurgical Engineering Department, Institut Teknologi Kalimantan, Soekarno-Hatta KM. 15, Balikpapan 76127, Indonesia

^4^ Department of Microbial Diseases, UCL Eastman Dental Institute, Royal Free Campus, University College London, London, United Kingdom

^5^ Department of Biochemistry and Genetics, Kagoshima University Graduate School of Medical and Dental Sciences, 8-35-1 Sakuragaoka, Kagoshima 890-8544, Japan

^6^ UCL Eastman Dental Institute, University College London, London, United Kingdom

^7^ NIHR UCLH Biomedical Research Centre, London, UK

^8^ Insigneo Institute, University of Sheffield, United Kingdom

**Supplementary Information**

**
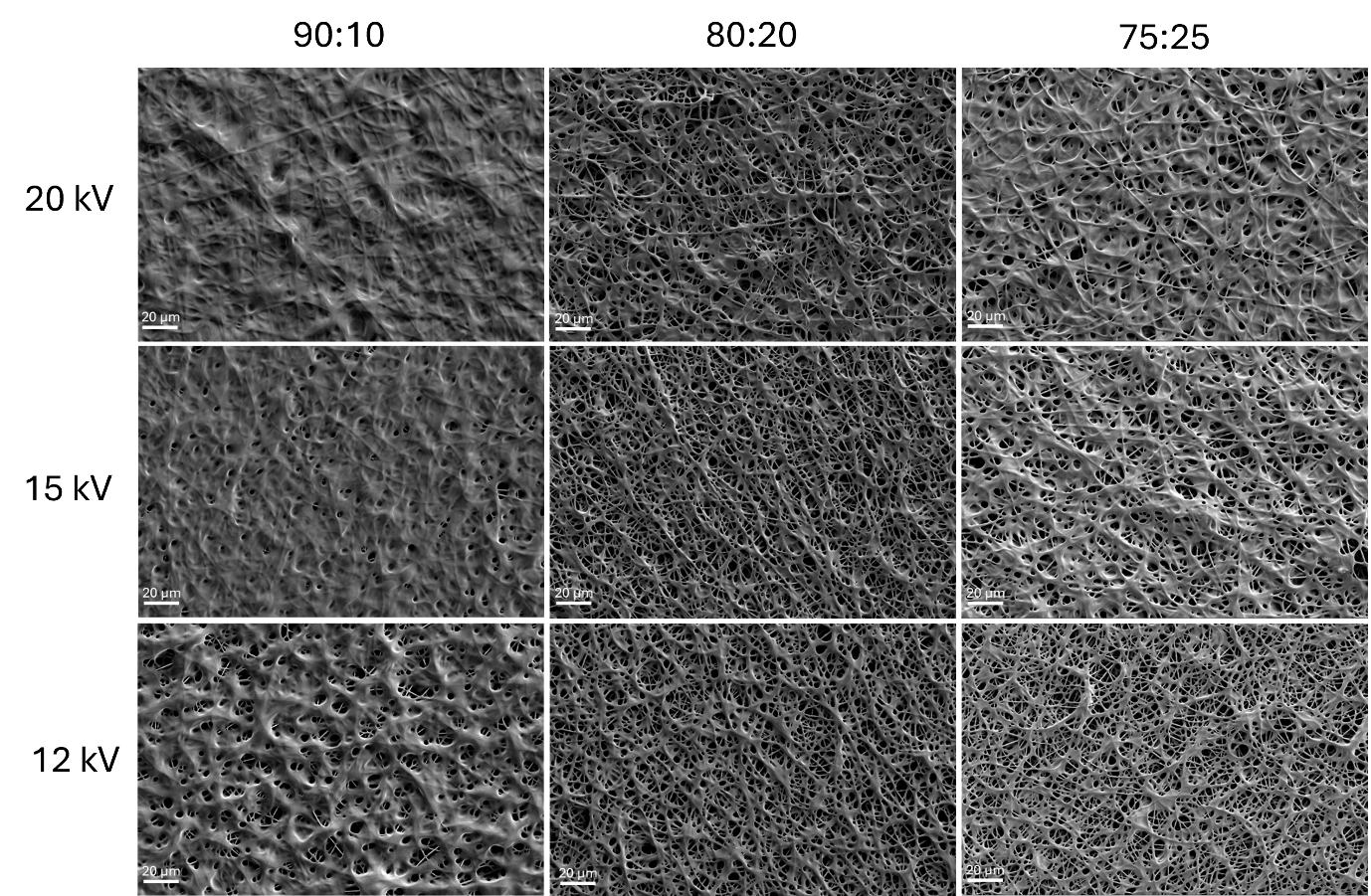
**

**SI1)** SEM images taken at magnification x200 of electrospun fibres fabricated using 5 wt% (w/v) of various MCL:SCL PHA blend solutions at a range of different applied voltages.


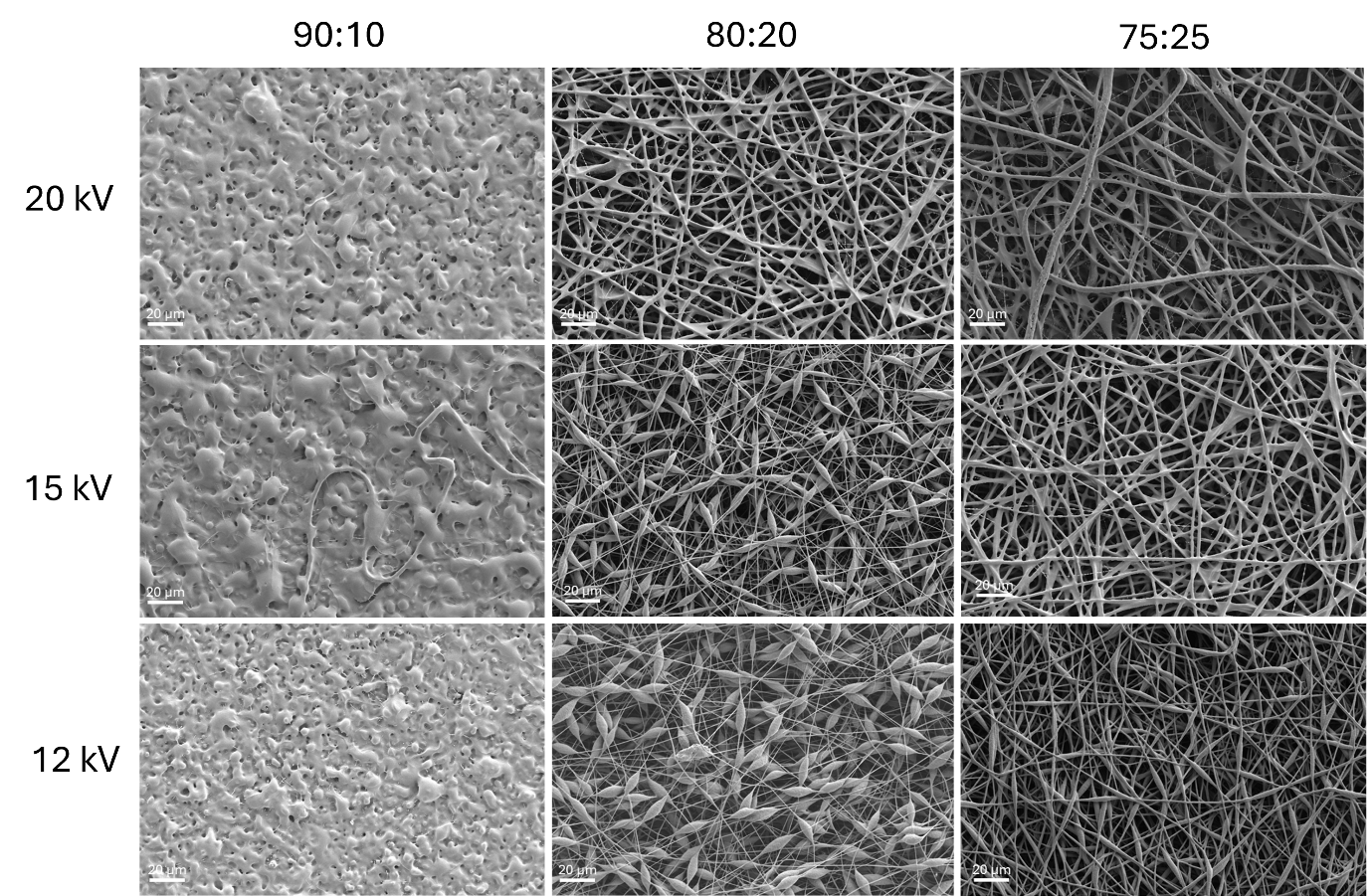


**SI2)** SEM images taken at magnification x200 of electrospun fibres fabricated using 10 wt% (w/v) of various MCL:SCL PHA blend solutions at a range of different applied voltages.

**SI3)** A summary of the physical properties of all sample groups which formed distinct fibrous structure, all analysis was done on 5 separate samples per group.

| **Sample Group** | **Representative SEM** | **Mean Diameter/µm** | **Standard Deviation/µm** | **Histogram of Fibre Diameters** | **Percentage Porosity/%** |
| --- | --- | --- | --- | --- | --- |
| 7525 @ 12kV 1 ml.hr^-1^ | 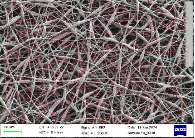 | 1.33 | 0.77 | 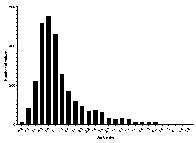 | 46.0 ± 0.6 |
| 7525 @ 20kV 1 ml.hr^-1^ | 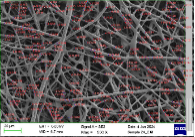 | 0.97 | 0.21 | 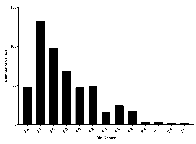 | 46.9 ± 0.4 |
| 7525 @ 15kV 0.75 ml.hr^-1^ | 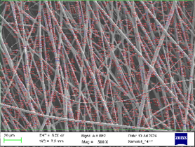 | 1.83 | 0.22 | 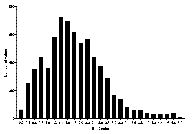 | 42.0 ± 4.8 |
| 7525 @ 15kV 1 ml.hr^-1^ | 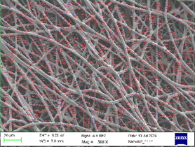 | 2.13 | 0.26 | 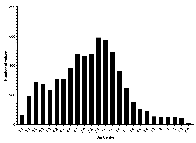 | 34.3 ± 0.7 |
| 8020 @ 12kV 1 ml.hr^-1^ | 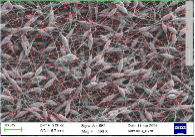 | 1.15 | 1.03 | 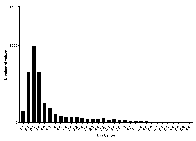 | 36.4 ± 2.5 |
| 8020 @ 15kV 1 ml.hr^-1^ | 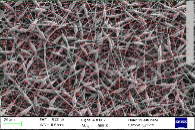 | 1.12 | 0.97 | 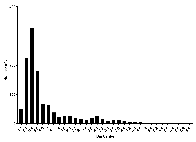 | 35.6 ± 0.8 |
| 8020 @ 20 kV 0.75 ml.hr^-1^ | 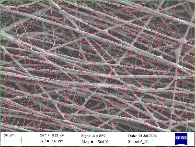 | 1.88 | 1.01 | 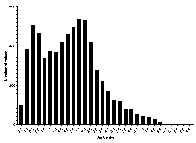 | 35.9 ± 3.8 |
| 8020 @ 20kV  1 ml.hr^-1^ | 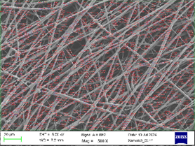 | 1.65 | 0.22 | 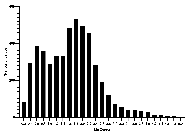 | 38.5 ± 1.2 |

**SI4)** Full raw data analysis of the mechanical properties of the sample during electrospinning optimisation, referred to as ES1-ES4 in the main manuscript, n=5.

| **Sample Group** | **Young’s Modulus ± SD /MPa** | **Tensile Strength ± SD /MPa** | **Maximum Strain ± SD / %** |
| --- | --- | --- | --- |
| **ES1** | 4.11 ± 0.22 | 3.54 ± 0.39 | 92.27 ± 13.35 |
| **ES2** | 2.65 ± 0.52 | 2.68 ± 0.23 | 85.80 ± 12.04 |
| **ES3** | 4.22 ± 0.30 | 2.69 ± 0.33 | 81.79 ± 8.54 |
| **ES4** | 3.21 ± 0.52 | 2.58 ± 0.53 | 71.61 ± 16.43 |


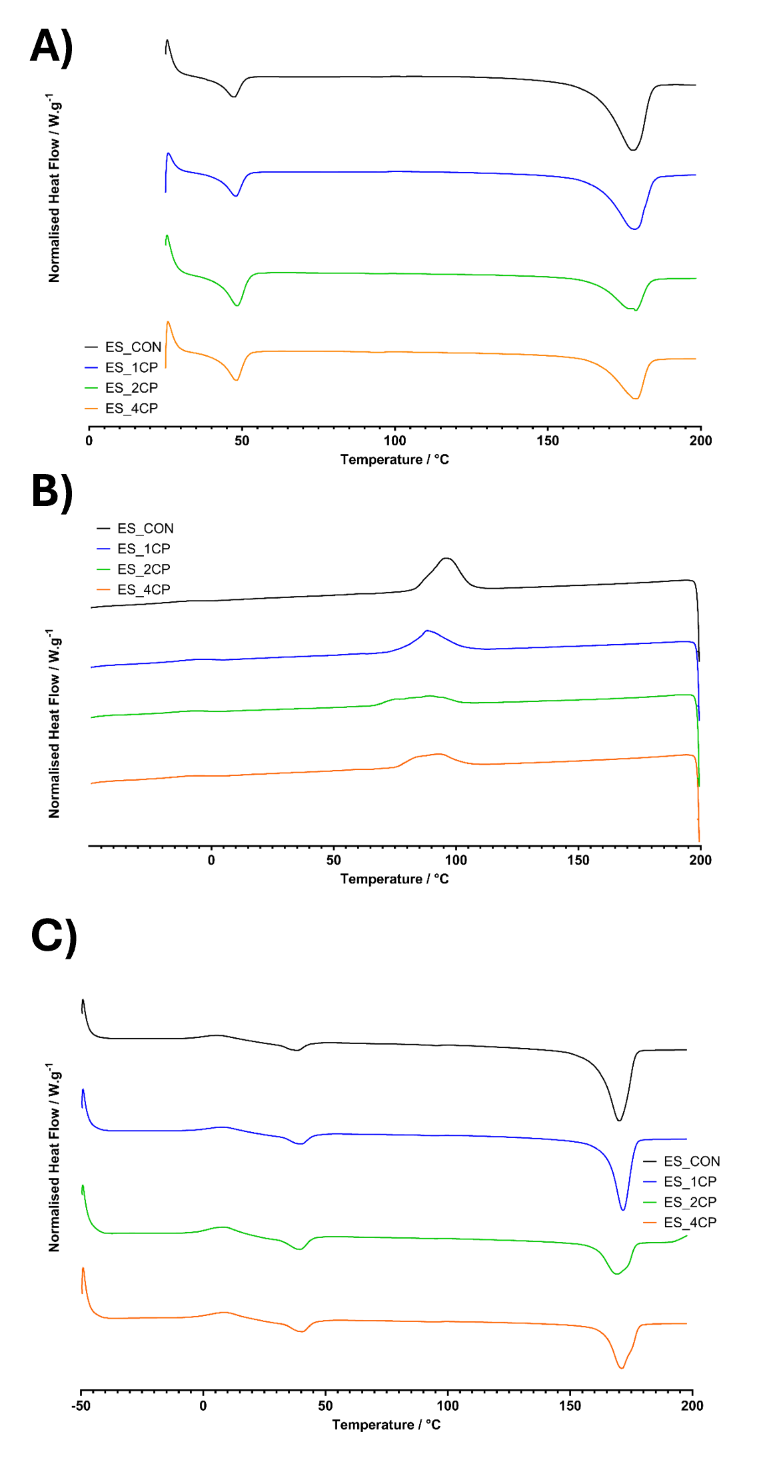


**SI5)** DSC thermograms showing the thermal events of electrospun MCL:SCL PHA blends at a ratio of 80:20 containing 0-4% CP.


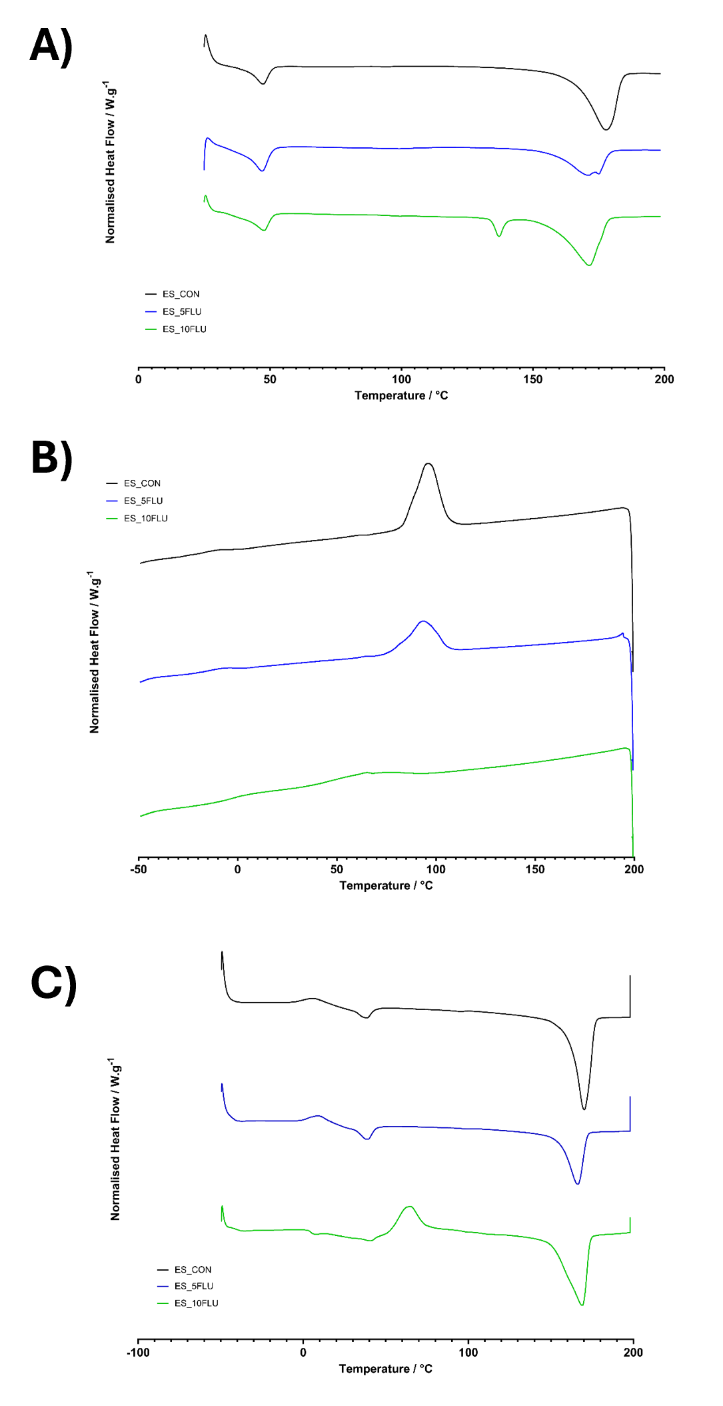


**SI6)** DSC thermograms showing the thermal events of electrospun MCL:SCL PHA blends at a ratio of 80:20 containing 0-10% FLU.


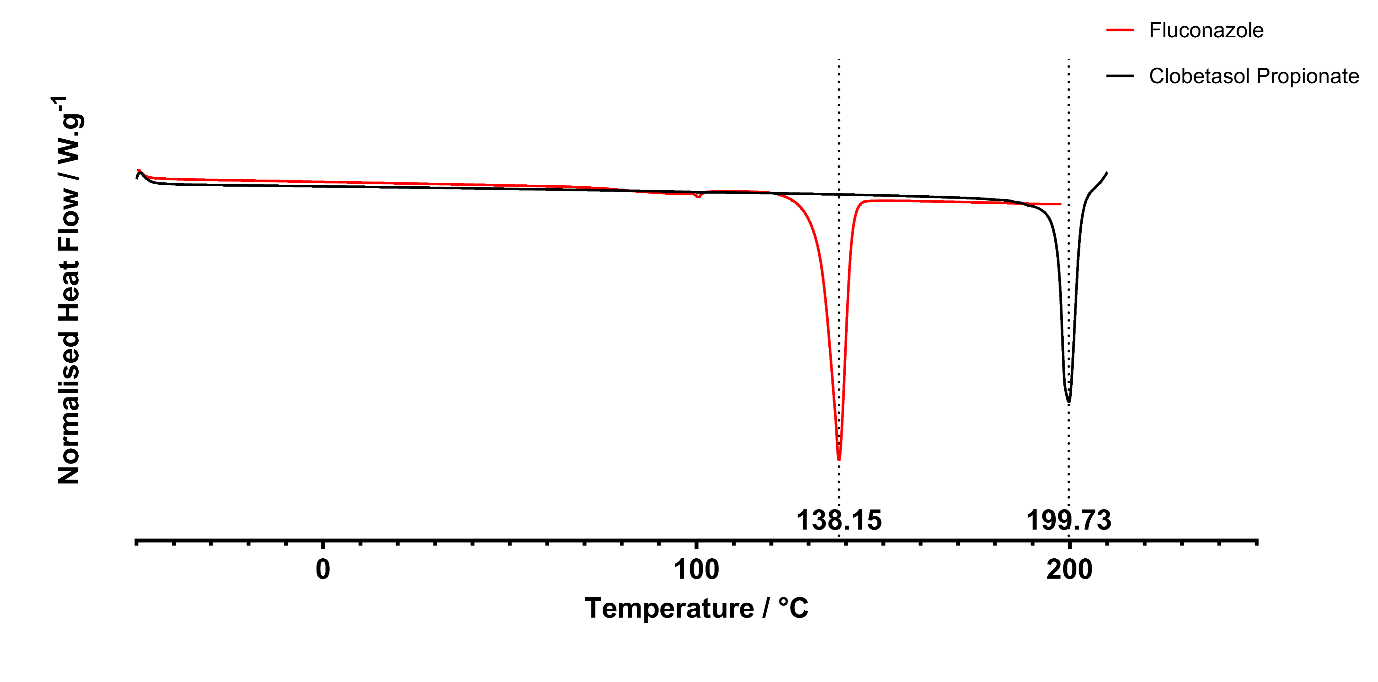


**SI7)** DSC thermograms showing the thermal events of the pure drug powders CP and FLU.


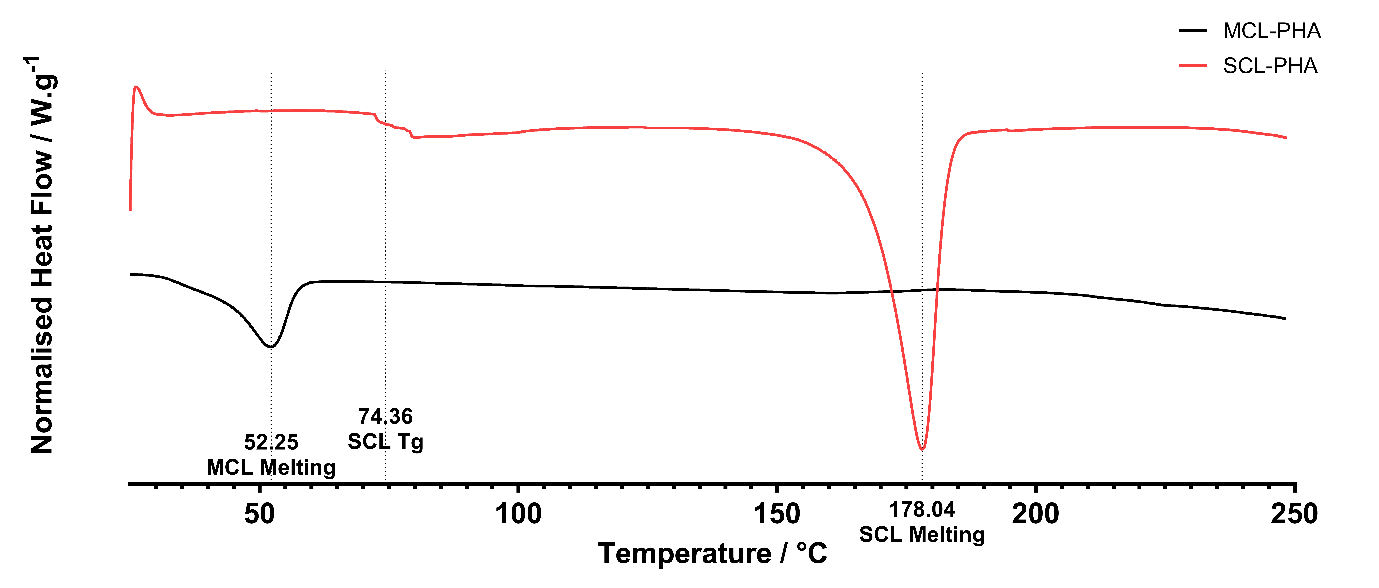


**SI8)** DSC thermograms showing the thermal events of the pure polymer, MCL-PHA and SCL-PHA.

**SI9)** Calculated regression coefficients of different mathematical models of drug release for each of the scaffold groups containing CP. Where Q= m_t_/m_0,_ experimentally obtained CP release up to time t. K is the kinetic constant of each model and n is the release exponent.

| **Material** | **Zeroth Order**  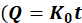) | **First Order**  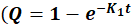**)** | **Higuchi Model** 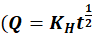**)** | **Korsmeyer-Peppas**  **(**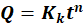**)** |
| --- | --- | --- | --- | --- |
| **ES_1CP** | 0.93 | 0.92 | 0.95 | 0.98 |
| **ES_2CP** | 0.63 | 0.92 | 0.88 | 0.96 |
| **ES_4CP** | 0.76 | 0.96 | 0.91 | 0.90 |

**SI10)** Calculated regression coefficients of different mathematical models of drug release for each of the scaffold groups containing FLU. Where Q= m_t_/m_0,_ experimentally obtained FLU release up to time t. K is the kinetic constant of each model and n is the release exponent.

| **Material** | **Zeroth Order**  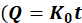) | **First Order**  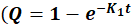**)** | **Higuchi Model** 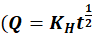**)** | **Korsmeyer-Peppas**  **(**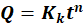**)** |
| --- | --- | --- | --- | --- |
| **ES_5FLU** | 0.67 | 0.94 | 0.92 | 0.95 |
| **ES_10FLU** | 0.65 | 0.92 | 0.91 | 0.98 |

**SI 11)** Detail on the calculated released mass of CP at each time point of the drug release study as a percentage of the maximum solubility of CP in the receptor phase. Where maximum solubility of CP in our release media under the conditions of the drug release study was calculated using the methodology below.

PBS was prepared at a pH of 7.4 and CP was added in excess at a concentration of 10 µg.mL^-1^ the solution was placed at 37°C under agitation at 100 rpm for 24 hours to ensure solubility equilibrium was reached. The samples were then filtered using a 0.45 µm syringe filter to remove any undissolved CP, then analysed using the HPLC method and the maximum solubility calculated.

The experimentally determined maximum solubility of CP was 4.498 µg.mL^-1^.

| **Time / Hours** | **Quantity of CP Released / µg.mL^-1^** | | | **Percentage of Solution Saturation / %** | | |
| --- | --- | --- | --- | --- | --- | --- |
|  | **ES_1CP** | **ES_2CP** | **ES_4CP** | **ES_1CP** | **ES_2CP** | **ES_4CP** |
| **0.5** | 0.328 | 0.736 | 0.413 | 7.301 | 16.360 | 9.185 |
| **1** | 0.112 | 0.272 | 0.296 | 2.499 | 6.052 | 6.576 |
| **2** | 0.053 | 0.266 | 0.336 | 1.175 | 5.916 | 7.469 |
| **3** | 0.046 | 0.254 | 0.332 | 1.020 | 5.649 | 7.391 |
| **4** | 0.041 | 0.278 | 0.307 | 0.917 | 6.192 | 6.834 |
| **5** | 0.264 | 0.513 | 1.075 | 5.871 | 11.401 | 23.891 |
| **6** | 0.259 | 0.426 | 1.017 | 5.754 | 9.467 | 22.607 |
| **8** | 0.238 | 0.477 | 1.003 | 5.297 | 10.599 | 22.291 |
| **12** | 0.232 | 0.354 | 0.920 | 5.160 | 7.864 | 20.466 |
| **24** | 0.165 | 0.225 | 0.476 | 3.669 | 4.997 | 10.587 |

**SI 12)** Detail on the calculated released mass of FLU at each time point of the drug release study as a percentage of the maximum solubility of FLU in the receptor phase. Where maximum solubility of FLU in our release media under the conditions of the drug release study was calculated using the methodology below.

PBS was prepared at a pH of 7.4 and FLU was added in excess at a concentration of 3 mg.mL^-1^ the solution was placed at 37°C under agitation at 100 rpm for 24 hours to ensure solubility equilibrium was reached. The sample was then filtered using a 0.45 µm syringe filter to remove any undissolved FLU. The resulting supernatant was diluted by a factor of 10 to ensure concentration fell within the linear range of the previously established calibration curve, then analysed using the HPLC method and the maximum solubility calculated.

The experimentally determined maximum solubility of FLU was 1.287 mg.mL^-1^.

| **Time / Hours** | **Quantity of FLU Released / µg.mL^-1^** | | **Percentage of Solution Saturation / %** | |
| --- | --- | --- | --- | --- |
|  | **ES_5FLU** | **ES_10FLU** | **ES_5FLU** | **ES_10FLU** |
| **0.5** | 30.087 | 56.531 | 2.338 | 4.392 |
| **1** | 29.633 | 52.673 | 2.302 | 4.092 |
| **2** | 26.011 | 49.649 | 2.021 | 3.857 |
| **3** | 24.755 | 46.971 | 1.923 | 3.649 |
| **4** | 20.148 | 49.074 | 1.565 | 3.813 |
| **5** | 18.433 | 48.039 | 1.432 | 3.732 |
| **6** | 17.646 | 47.283 | 1.371 | 3.674 |
| **8** | 22.606 | 42.880 | 1.756 | 3.332 |
| **12** | 24.741 | 41.814 | 1.922 | 3.249 |
| **24** | 20.725 | 34.931 | 1.610 | 2.714 |


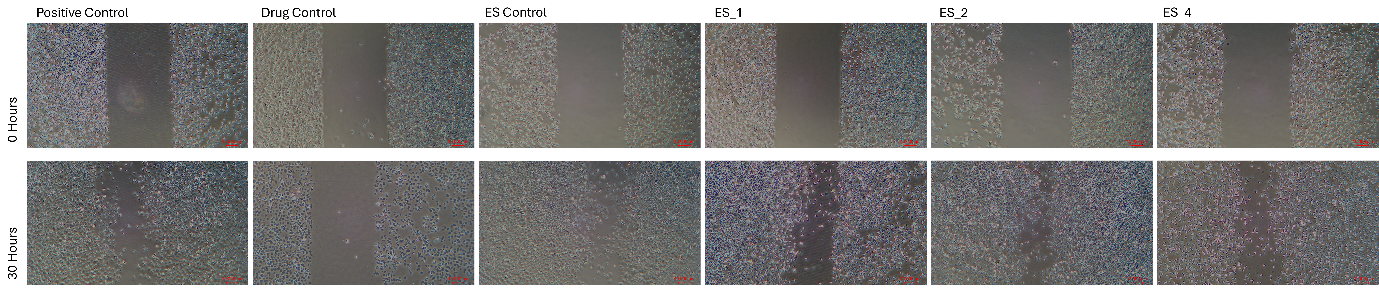


**SI13)** Phase contrast images showing the wound area at 0 hours and 30 hours following treatment with the CP control and electrospun dosage forms.

**
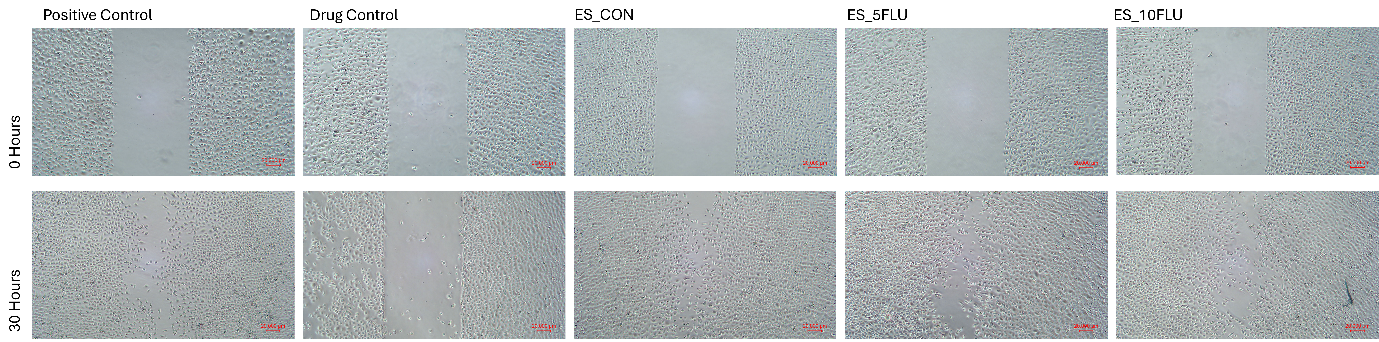
**

**S14)** Phase contrast images showing the wound area at 0 hours and 30 hours following treatment with the FLU control and electrospun dosage forms.

**SI15)** P values corresponding to the statistical analyses conducted throughout the manuscript, where the significance displayed on graphs is represented by asterisks, where * represents P≤0.05, ** represents P≤0.01, *** represents P≤0.001 and **** represents P≤0.0001.

**P-values corresponding to the Young’s modulus analysis of the electrospun scaffold groups during optimisation shown in Figure 2B.**

| Sample Group I | Sample Group II | P-value |
| --- | --- | --- |
| ES1 | ES2 | 0.0003 |
| ES1 | ES3 | 0.9778 |
| ES1 | ES4 | 0.0187 |
| ES2 | ES3 | 0.0003 |
| ES2 | ES4 | 0.1988 |
| ES3 | ES4 | 0.0126 |

**P-values corresponding to the fibre diameter analysis of the CP loaded electrospun scaffold groups during optimisation shown in Figure 3E.**

| Sample Group I | Sample Group II | P-value |
| --- | --- | --- |
| ES_CON | ES_1CP | 0.4713 |
| ES_CON | ES_2CP | 0.8418 |
| ES_CON | ES_4CP | 0.9771 |
| ES_1CP | ES_2CP | 0.9725 |
| ES_1CP | ES_4CP | 0.8106 |
| ES_2CP | ES_4CP | 0.9977 |

**P-values corresponding to the Young’s modulus analysis of the CP loaded electrospun scaffold groups during optimisation shown in Figure 3G.**

| Testing Method | Sample Group I | Sample Group II | P-value |
| --- | --- | --- | --- |
| 21°C | ES_CON | ES_1CP | 0.6382 |
| 21°C | ES_CON | ES_2CP | 0.9971 |
| 21°C | ES_CON | ES_4CP | 0.0250 |
| 21°C | ES_1CP | ES_2CP | 0.6316 |
| 21°C | ES_1CP | ES_4CP | 0.0415 |
| 21°C | ES_2CP | ES_4CP | 0.0940 |
| 37°C | ES_CON | ES_1CP | 0.2003 |
| 37°C | ES_CON | ES_2CP | 0.5388 |
| 37°C | ES_CON | ES_4CP | 0.0621 |
| 37°C | ES_1CP | ES_2CP | 0.0603 |
| 37°C | ES_1CP | ES_4CP | 0.9377 |
| 37°C | ES_2CP | ES_4CP | 0.0214 |
| 37°C (submerged) | ES_CON | ES_1CP | 0.8598 |
| 37°C (submerged) | ES_CON | ES_2CP | >0.9999 |
| 37°C (submerged) | ES_CON | ES_4CP | 0.9739 |
| 37°C (submerged) | ES_1CP | ES_2CP | 0.4421 |
| 37°C (submerged) | ES_1CP | ES_4CP | 0.1492 |
| 37°C (submerged) | ES_2CP | ES_4CP | 0.7855 |

**P-values corresponding to the fibre diameter analysis of the FLU loaded electrospun scaffold groups during optimisation shown in Figure 4D.**

| Sample Group I | Sample Group II | P-value |
| --- | --- | --- |
| ES_CON | ES_5FLU | 0.9765 |
| ES_CON | ES_10FLU | 0.4370 |
| ES_5FLU | ES_10FLU | 0.6638 |

**P-values corresponding to the Young’s modulus analysis of the FLU loaded electrospun scaffold groups during optimisation shown in Figure 4F.**

| Testing Method | Sample Group I | Sample Group II | P-value |
| --- | --- | --- | --- |
| 21°C | ES_CON | ES_5FLU | 0.0137 |
| 21°C | ES_CON | ES_1-FLU | 0.0259 |
| 21°C | ES_5FLU | ES_10FLU | 0.1010 |
| 37°C | ES_CON | ES_5FLU | 0.0690 |
| 37°C | ES_CON | ES_1-FLU | 0.0121 |
| 37°C | ES_5FLU | ES_10FLU | 0.6953 |
| 37°C (submerged) | ES_CON | ES_5FLU | 0.0744 |
| 37°C (submerged) | ES_CON | ES_1-FLU | 0.0049 |
| 37°C (submerged) | ES_5FLU | ES_10FLU | 0.2602 |

**P-values corresponding to the MOE-1a viability analysis of the CP loaded electrospun scaffold groups during optimisation shown in Figure 6A.**

| Study Time | Sample Group I | Sample Group II | P-value |
| --- | --- | --- | --- |
| Day 1 | Pos Con | Drug Con | <0.0001 |
| Day 1 | Pos Con | ES_CON | 0.9462 |
| Day 1 | Pos Con | ES_1CP | 0.7123 |
| Day 1 | Pos Con | ES_2CP | 0.1076 |
| Day 1 | Pos Con | ES_4CP | 0.0209 |
| Day 1 | Drug Con | ES_CON | <0.0001 |
| Day 1 | Drug Con | ES_1CP | <0.0001 |
| Day 1 | Drug Con | ES_2CP | <0.0001 |
| Day 1 | Drug Con | ES_4CP | <0.0001 |
| Day 1 | ES_CON | ES_1CP | 0.9775 |
| Day 1 | ES_CON | ES_2CP | 0.1586 |
| Day 1 | ES_CON | ES_4CP | 0.0236 |
| Day 1 | ES_1 | ES_2CP | >0.9999 |
| Day 1 | ES_1 | ES_4CP | 0.8488 |
| Day 1 | ES_2 | ES_4CP | 0.8077 |
| Day 3 | Pos Con | Drug Con | <0.0001 |
| Day 3 | Pos Con | ES_CON | 0.9875 |
| Day 3 | Pos Con | ES_1CP | 0.7326 |
| Day 3 | Pos Con | ES_2CP | 0.2008 |
| Day 3 | Pos Con | ES_4CP | 0.9855 |
| Day 3 | Drug Con | ES_CON | <0.0001 |
| Day 3 | Drug Con | ES_1CP | <0.0001 |
| Day 3 | Drug Con | ES_2CP | <0.0001 |
| Day 3 | Drug Con | ES_4CP | <0.0001 |
| Day 3 | ES_CON | ES_1CP | >0.9999 |
| Day 3 | ES_CON | ES_2CP | 0.8212 |
| Day 3 | ES_CON | ES_4CP | >0.9999 |
| Day 3 | ES_1 | ES_2CP | 0.9561 |
| Day 3 | ES_1 | ES_4CP | >0.9999 |
| Day 3 | ES_2 | ES_4CP | 0.8372 |
| Day 7 | Pos Con | Drug Con | <0.0001 |
| Day 7 | Pos Con | ES_CON | >0.9999 |
| Day 7 | Pos Con | ES_1CP | 0.9770 |
| Day 7 | Pos Con | ES_2CP | 0.5311 |
| Day 7 | Pos Con | ES_4CP | 0.0994 |
| Day 7 | Drug Con | ES_CON | <0.0001 |
| Day 7 | Drug Con | ES_1CP | <0.0001 |
| Day 7 | Drug Con | ES_2CP | <0.0001 |
| Day 7 | Drug Con | ES_4CP | <0.0001 |
| Day 7 | ES_CON | ES_1CP | 0.9870 |
| Day 7 | ES_CON | ES_2CP | 0.4129 |
| Day 7 | ES_CON | ES_4CP | 0.0519 |
| Day 7 | ES_1 | ES_2CP | >0.9999 |
| Day 7 | ES_1 | ES_4CP | 0.9503 |
| Day 7 | ES_2 | ES_4CP | 0.8078 |

**P-values corresponding to the MOE-1a wound healing analysis of the CP loaded electrospun scaffold groups during optimisation shown in Figure 6C.**

| Study Time | Sample Group I | Sample Group II | P-value |
| --- | --- | --- | --- |
| 12 hours | Pos Con | Drug Con | 0.2719 |
| 12 hours | Pos Con | ES_CON | >0.9999 |
| 12 hours | Pos Con | ES_1CP | >0.9999 |
| 12 hours | Pos Con | ES_2CP | 0.1312 |
| 12 hours | Pos Con | ES_4CP | 0.9863 |
| 12 hours | Drug Con | ES_CON | 0.2805 |
| 12 hours | Drug Con | ES_1CP | 0.0630 |
| 12 hours | Drug Con | ES_2CP | 0.0058 |
| 12 hours | Drug Con | ES_4CP | 0.1069 |
| 12 hours | ES_CON | ES_1CP | >0.9999 |
| 12 hours | ES_CON | ES_2CP | 0.1290 |
| 12 hours | ES_CON | ES_4CP | 0.9841 |
| 12 hours | ES_1CP | ES_2CP | 0.0749 |
| 12 hours | ES_1CP | ES_4CP | 0.9989 |
| 12 hours | ES_2CP | ES_4CP | 0.4231 |
| 24 hours | Pos Con | Drug Con | <0.0001 |
| 24 hours | Pos Con | ES_CON | 0.3638 |
| 24 hours | Pos Con | ES_1CP | 0.9995 |
| 24 hours | Pos Con | ES_2CP | 0.4677 |
| 24 hours | Pos Con | ES_4CP | >0.9999 |
| 24 hours | Drug Con | ES_CON | 0.0010 |
| 24 hours | Drug Con | ES_1CP | 0.0015 |
| 24 hours | Drug Con | ES_2CP | 0.0077 |
| 24 hours | Drug Con | ES_4CP | 0.0384 |
| 24 hours | ES_CON | ES_1CP | 0.4581 |
| 24 hours | ES_CON | ES_2CP | 0.1322 |
| 24 hours | ES_CON | ES_4CP | 0.9695 |
| 24 hours | ES_1CP | ES_2CP | 0.8220 |
| 24 hours | ES_1CP | ES_4CP | >0.9999 |
| 24 hours | ES_2CP | ES_4CP | 0.8755 |
| 30 hours | Pos Con | Drug Con | <0.0001 |
| 30 hours | Pos Con | ES_CON | >0.9999 |
| 30 hours | Pos Con | ES_1CP | 0.8035 |
| 30 hours | Pos Con | ES_2CP | 0.0003 |
| 30 hours | Pos Con | ES_4CP | 0.4297 |
| 30 hours | Drug Con | ES_CON | 0.0004 |
| 30 hours | Drug Con | ES_1CP | 0.0004 |
| 30 hours | Drug Con | ES_2CP | <0.0001 |
| 30 hours | Drug Con | ES_4CP | 0.0048 |
| 30 hours | ES_CON | ES_1CP | 0.9594 |
| 30 hours | ES_CON | ES_2CP | 0.0346 |
| 30 hours | ES_CON | ES_4CP | 0.4745 |
| 30 hours | ES_1CP | ES_2CP | 0.1347 |
| 30 hours | ES_1CP | ES_4CP | 0.2168 |
| 30 hours | ES_2CP | ES_4CP | 0.0471 |

**P-values corresponding to the MOE-1a viability analysis of the FLU loaded electrospun scaffold groups during optimisation shown in Figure 7A.**

| Study Time | Sample Group I | Sample Group II | P-value |
| --- | --- | --- | --- |
| Day 1 | Pos Con | Drug Con | <0.0001 |
| Day 1 | Pos Con | ES_CON | 0.2456 |
| Day 1 | Pos Con | ES_5FLU | 0.3755 |
| Day 1 | Pos Con | ES_10FLU | 0.6891 |
| Day 1 | Drug Con | ES_CON | <0.0001 |
| Day 1 | Drug Con | ES_5FLU | <0.0001 |
| Day 1 | Drug Con | ES_10FLU | <0.0001 |
| Day 1 | ES_CON | ES_5FLU | 0.7767 |
| Day 1 | ES_CON | ES_10FLU | 0.4402 |
| Day 1 | ES_5FLU | ES_10FLU | 0.6231 |
| Day 3 | Pos Con | Drug Con | 0.0648 |
| Day 3 | Pos Con | ES_CON | 0.9915 |
| Day 3 | Pos Con | ES_5FLU | 0.1829 |
| Day 3 | Pos Con | ES_10FLU | 0.9090 |
| Day 3 | Drug Con | ES_CON | 0.1561 |
| Day 3 | Drug Con | ES_5FLU | 0.0003 |
| Day 3 | Drug Con | ES_10FLU | 0.0091 |
| Day 3 | ES_CON | ES_5FLU | 0.0777 |
| Day 3 | ES_CON | ES_10FLU | 0.6924 |
| Day 3 | ES_5FLU | ES_10FLU | 0.6172 |
| Day 7 | Pos Con | Drug Con | <0.0001 |
| Day 7 | Pos Con | ES_CON | 0.7767 |
| Day 7 | Pos Con | ES_5FLU | 0.9997 |
| Day 7 | Pos Con | ES_10FLU | 0.0178 |
| Day 7 | Drug Con | ES_CON | <0.0001 |
| Day 7 | Drug Con | ES_5FLU | <0.0001 |
| Day 7 | Drug Con | ES_10FLU | <0.0001 |
| Day 7 | ES_CON | ES_5FLU | 0.8650 |
| Day 7 | ES_CON | ES_10FLU | 0.1982 |
| Day 7 | ES_5FLU | ES_10FLU | 0.0271 |

**P-values corresponding to the MOE-1a wound healing analysis of the FLU loaded electrospun scaffold groups during optimisation shown in Figure 7C.**

| Study Time | Sample Group I | Sample Group II | P-value |
| --- | --- | --- | --- |
| 12 hours | Pos Con | Drug Con | 0.2719 |
| 12 hours | Pos Con | ES_CON | >0.9999 |
| 12 hours | Pos Con | ES_1CP | >0.9999 |
| 12 hours | Pos Con | ES_2CP | 0.1312 |
| 12 hours | Pos Con | ES_4CP | 0.9863 |
| 12 hours | Drug Con | ES_CON | 0.2805 |
| 12 hours | Drug Con | ES_1CP | 0.0630 |
| 12 hours | Drug Con | ES_2CP | 0.0058 |
| 12 hours | Drug Con | ES_4CP | 0.1069 |
| 12 hours | ES_CON | ES_1CP | >0.9999 |
| 12 hours | ES_CON | ES_2CP | 0.1290 |
| 12 hours | ES_CON | ES_4CP | 0.9841 |
| 12 hours | ES_1CP | ES_2CP | 0.0749 |
| 12 hours | ES_1CP | ES_4CP | 0.9989 |
| 12 hours | ES_2CP | ES_4CP | 0.4231 |
| 24 hours | Pos Con | Drug Con | <0.0001 |
| 24 hours | Pos Con | ES_CON | 0.3638 |
| 24 hours | Pos Con | ES_1CP | 0.9995 |
| 24 hours | Pos Con | ES_2CP | 0.4677 |
| 24 hours | Pos Con | ES_4CP | >0.9999 |
| 24 hours | Drug Con | ES_CON | 0.0010 |
| 24 hours | Drug Con | ES_1CP | 0.0015 |
| 24 hours | Drug Con | ES_2CP | 0.0077 |
| 24 hours | Drug Con | ES_4CP | 0.0384 |
| 24 hours | ES_CON | ES_1CP | 0.4581 |
| 24 hours | ES_CON | ES_2CP | 0.1322 |
| 24 hours | ES_CON | ES_4CP | 0.9695 |
| 24 hours | ES_1CP | ES_2CP | 0.8220 |
| 24 hours | ES_1CP | ES_4CP | >0.9999 |
| 24 hours | ES_2CP | ES_4CP | 0.8755 |
| 30 hours | Pos Con | Drug Con | <0.0001 |
| 30 hours | Pos Con | ES_CON | >0.9999 |
| 30 hours | Pos Con | ES_1CP | 0.8035 |
| 30 hours | Pos Con | ES_2CP | 0.0003 |
| 30 hours | Pos Con | ES_4CP | 0.4297 |
| 30 hours | Drug Con | ES_CON | 0.0004 |
| 30 hours | Drug Con | ES_1CP | 0.0004 |
| 30 hours | Drug Con | ES_2CP | <0.0001 |
| 30 hours | Drug Con | ES_4CP | 0.0048 |
| 30 hours | ES_CON | ES_1CP | 0.9594 |
| 30 hours | ES_CON | ES_2CP | 0.0346 |
| 30 hours | ES_CON | ES_4CP | 0.4745 |
| 30 hours | ES_1CP | ES_2CP | 0.1347 |
| 30 hours | ES_1CP | ES_4CP | 0.2168 |
| 30 hours | ES_2CP | ES_4CP | 0.0471 |
